# Supplementary material for: A ligand-insensitive UNC5B splicing isoform regulates angiogenesis by promoting apoptosis
Source: Nat Commun. 2021 Aug 11;12:4872. doi: 10.1038/s41467-021-24998-6 (PMC8358048; doi:10.1038/s41467-021-24998-6)
Supplement: Supplementary file 1 — Supplementary Information [file 41467_2021_24998_MOESM1_ESM.pdf]

Supplementary information

A ligand-insensitive UNC5B splicing isoform regulates angiogenesis by promoting apoptosis.

Pradella et al.,

Supplementary Figure 1

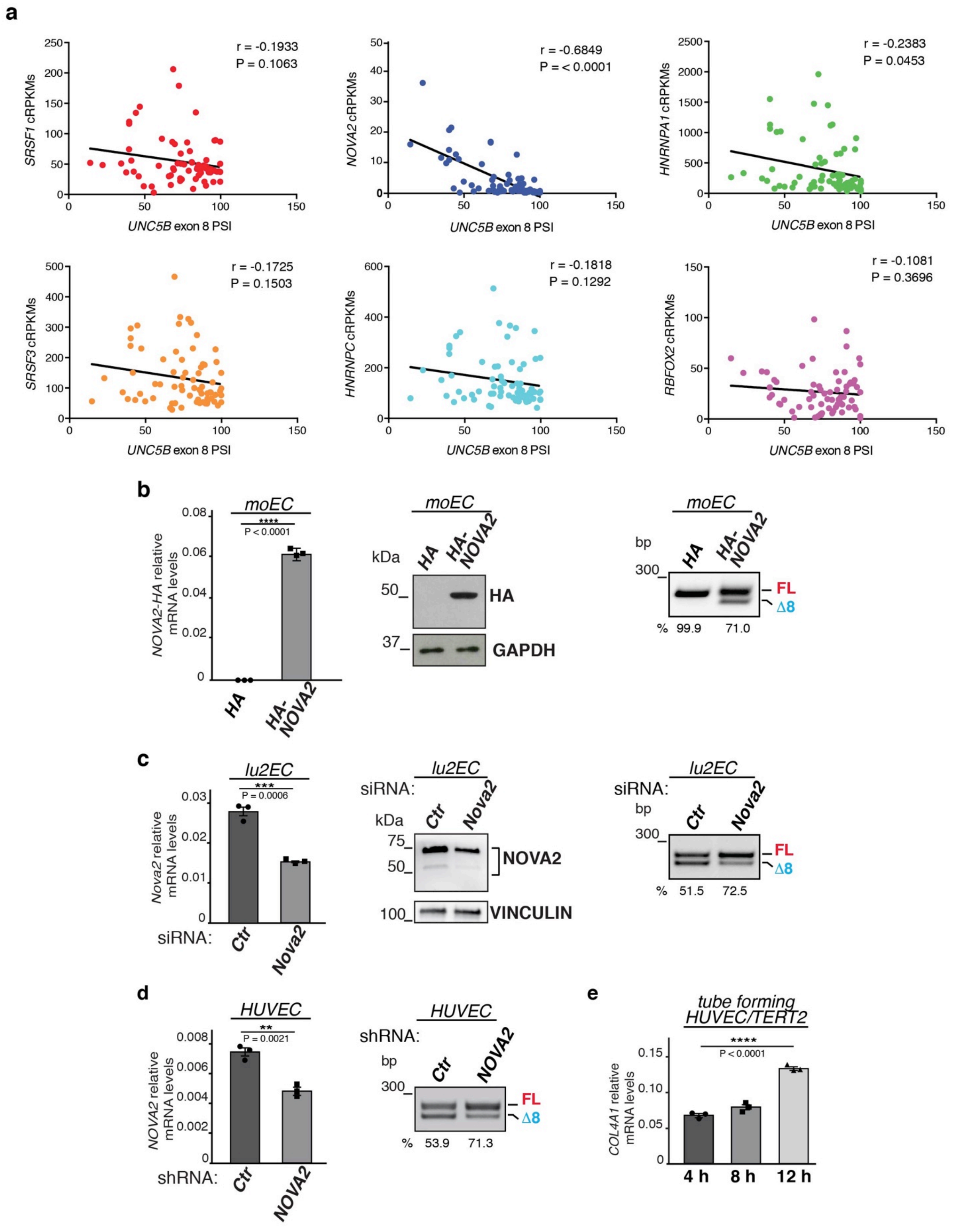

Supplementary Fig. 1. ***UNC5B* exon 8 inclusion in VastDB and its NOVA2-mediated regulation in ECs**. **a** *UNC5B* exon 8 PSI (Percent Spliced-In) correlation with several SRFs − including *SRSF1* (red), *NOVA2* (blue), *HNRNPA1* (green), *SRSF3* (orange), *HNRNPC* (cyan), or *RBFOX2* (magenta) − cRPKM expression values annotated in the Vertebrate Alternative Splicing and Transcription Database (VastDB; http://vastdb.crg.eu/). Pearson r values, associated P-values, and linear regression (black line) are shown. **b** Left: analysis by RT-qPCR (relative to *Ubb*) and

immunoblotting of NOVA2 HA-tagged expression levels in NOVA2 overexpressing moEC; GAPDH as loading control. Right: analysis by RT-PCR of *Unc5b* exon 8 splicing profile in the above ECs. n=3. **c** Left: analysis by RT-qPCR (relative to *Ubb*) and immunoblotting of NOVA2 expression levels in NOVA2-depleted lu2EC; VINCULIN as loading control. Right: analysis by RT-PCR of *Unc5b* exon 8 AS profile in the above ECs. n=3. **d** *NOVA2* mRNA levels (relative to *ACTB*) and AS analysis of *UNC5B* exon 8 in human primary ECs (HUVEC) stably expressing a pool of shRNAs against *NOVA2* or with a control shRNA pool. n=3. **e** *COL4A1* mRNA expression levels (relative to *B2M*) during *in vitro* angiogenesis of HUVEC/TERT2 plated on Matrigel-coated culture plates. h = hours after seeding. n=3. The percentage of exon inclusion is shown below each gel. Two-tailed Student's t-test or one-way ANOVA for multiple comparison; Error bars indicate ±SEM. Exact P-values (P) are indicated: ** < 0.01; *** < 0.001; **** < 0.0001.

Supplementary Figure 2

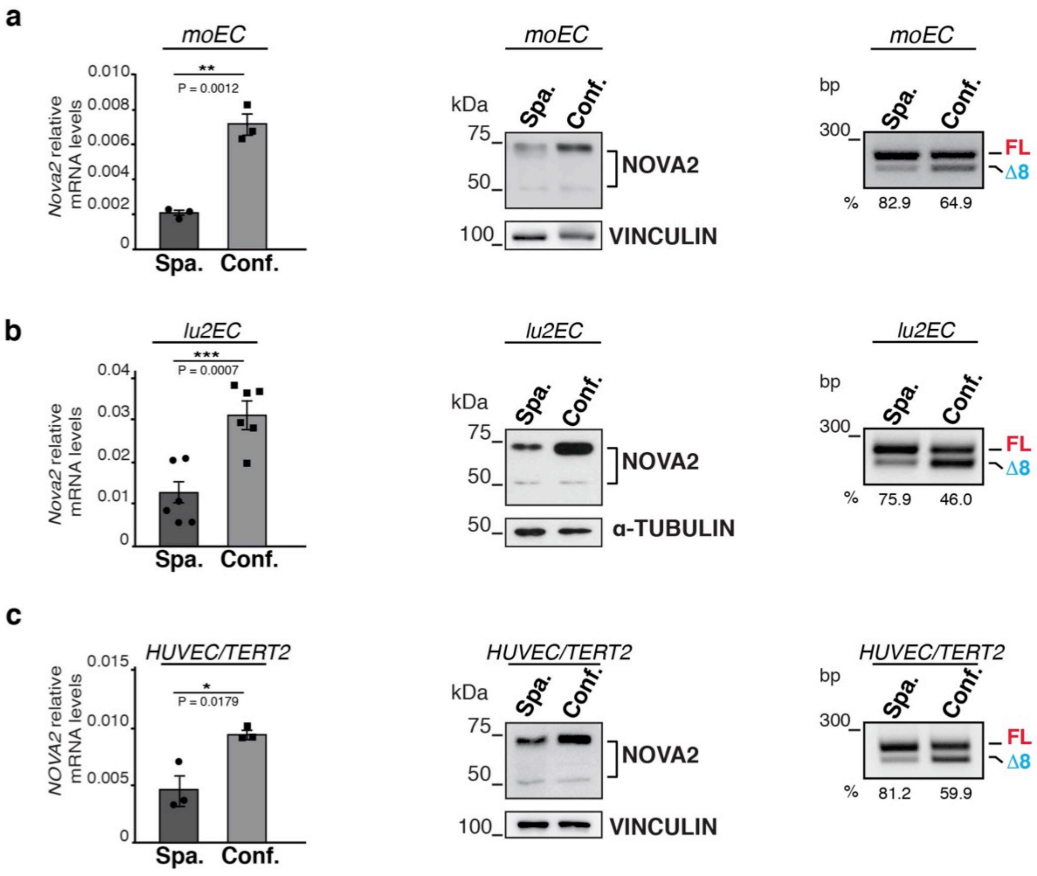

Supplementary Fig. 2. *UNC5B* exon 8 splicing and NOVA2 expression levels are regulated by cell density in ECs. Left: NOVA2 expression levels in moEC (**a**; n=3), lu2EC (**b**; n=6), and HUVEC/TERT2 (**c**; n=3) grown at different densities (sparse: Spa.; confluent: Conf.) were analyzed by RT-qPCR (relative to mouse *Ubb* or human *ACTB*) and immunoblotting. VINCULIN or α-TUBULIN as loading controls. Right: RT-PCR analysis of *UNC5B* exon 8 AS profiles in the above ECs grown as sparse (Spa.) or confluent (Conf.). The percentage of exon inclusion is shown below each gel. Two-tailed Student's t-test; Error bars indicate ±SEM. Exact P-values (P) are indicated: * < 0.05; ** < 0.01; *** < 0.001.

Supplementary Figure 3

a

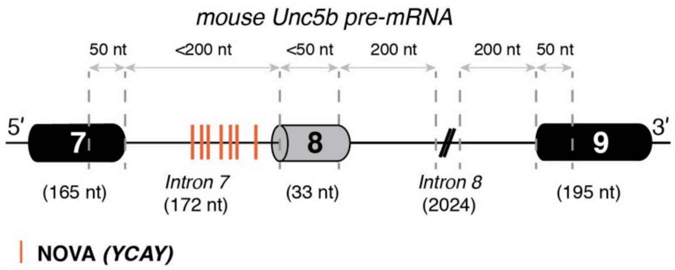

RBPmap

#	RBP	Motif	Z-score	P-value	Posit.
1	NOVA	ycay	3.920	4.43e-05	82 nt
2	NOVA	ycay	3.920	4.43e-05	87 nt
3	NOVA	ycay	3.920	4.43e-05	91 nt
4	NOVA	ycay	3.920	4.43e-05	96 nt
5	NOVA	ycay	3.920	4.43e-05	104 nt
6	NOVA	ycay	3.920	4.43e-05	107 nt
7	NOVA	ycay	3.840	6.15e-05	119 nt
8	PCBP3	uuuycc	3.784	7.72e-05	2198 nt
9	SRSF3	cuckucy	3.740	9.20e-05	226 nt
10	RBM24	wgwgugd	3.714	1.02e-04	2056 nt
11	PCBP3	uuuycc	3.676	1.18e-04	149 nt
12	PCBP3	uuuycc	3.676	1.18e-04	2175 nt
13	U2AF2	uuuuuyc	3.648	1.32e-04	306 nt
14	SRSF3	cuckucy	3.630	1.42e-04	233 nt
15	MBNL1	ygcuky	3.618	1.48e-04	352 nt

b

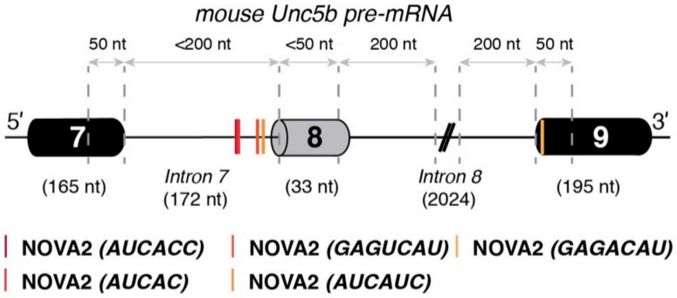

SpliceAid2

Protein	Recognized sequence	Binding Assay	Posit.
NOVA2	aucacc	SELEX	106 nt
NOVA2	aucac	X-ray crystallography	106 nt
NOVA2	gagucau	SELEX	139 nt
NOVA2	aucauc	SELEX	144 nt
NOVA2	gagacau	SELEX	2235 nt

c

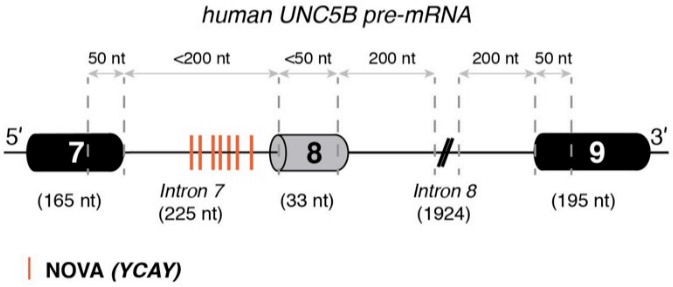

RBPmap

#	RBP	Motif	Z-score	P-value	Posit.
1	A1CF	ccwwhcc	4.260	1.02e-05	176 nt
2	A1CF	uuuycc	4.108	2.00e-05	202 nt
3	A1CF	uuuycc	4.095	2.11e-05	177 nt
4	A1CF	ccawmcc	4.072	2.33e-05	61 nt
5	A1CF	ccwwhcc	4.039	2.68e-05	152 nt
6	A1CF	ccawmcc	3.971	3.58e-05	62 nt
7	NOVA	ycay	3.960	3.75e-05	148 nt
8	NOVA	ycay	3.920	4.43e-05	135 nt
9	NOVA	ycay	3.920	4.43e-05	138 nt
10	NOVA	ycay	3.920	4.43e-05	143 nt
11	A1CF	ccawmcc	3.884	5.14e-05	73 nt
12	NOVA	ycay	3.840	6.15e-05	126 nt
13	NOVA	ycay	3.840	6.15e-05	129 nt
14	NOVA	ycay	3.840	6.15e-05	157 nt
15	A1CF	wcwwc	3.765	8.33e-05	143 nt

Supplementary Fig. 3. Prediction of NOVA2 binding motifs in *Unc5b* pre-mRNA. a Scheme of the *Unc5b* mouse genomic region analyzed with the RBPmap program. NOVA2 binding motifs are indicated as vertical orange bars. Right: top 15 predicted RNA binding motifs and their relative position to the *Unc5b* exon7-intron7 junction. **b** Prediction of RNA binding motifs in *Unc5b* mouse genomic region by using SpliceAid 2. **c** Scheme of the *UNC5B* human genomic region analyzed with the RBPmap program. NOVA2 binding motifs are indicated as vertical orange bars. Right: top 15 predicted RNA binding motifs and their relative position to the *UNC5B* exon7-intron7 junction. For RBPmap and SpliceAid 2 analyses, recognized motifs, and relative position to the *Unc5b* exon7-intron7 junction are also shown. Statistical parameters, calculated by RBPmap tool (Z-score and P-value), are also shown.

Supplementary Figure 4

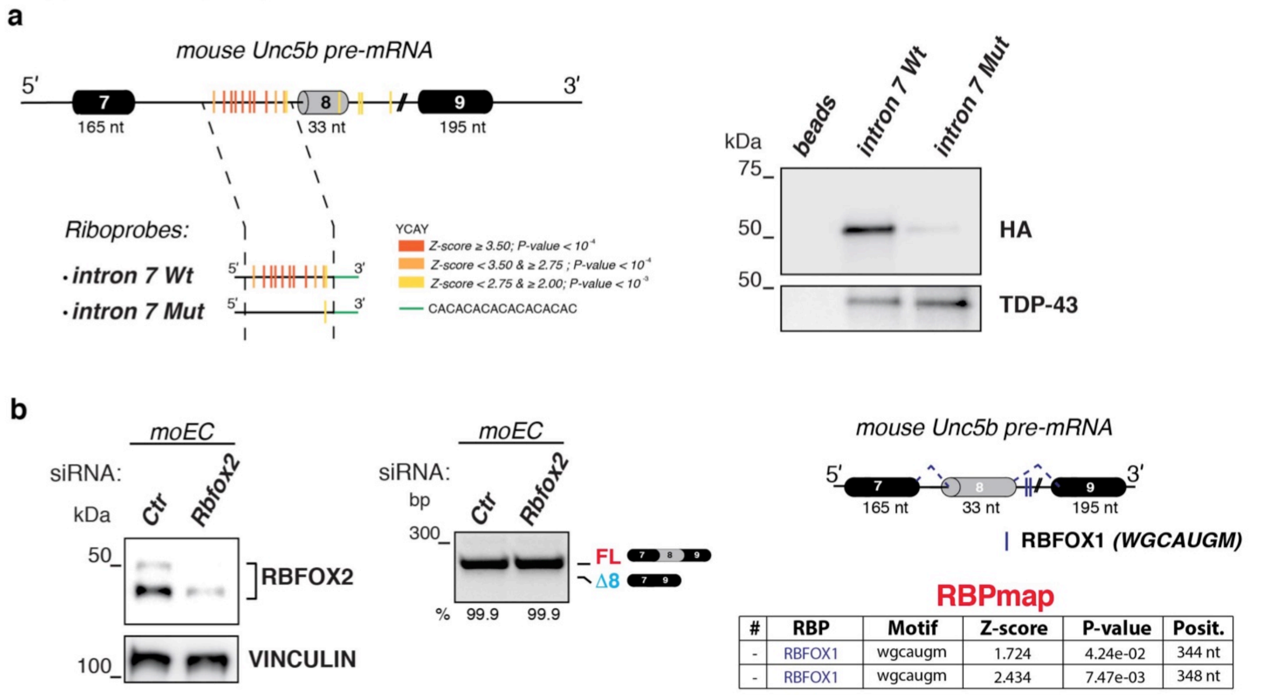

Supplementary Fig. 4. NOVA2 specifically recognizes the YCAY cluster in *Unc5b* intron 7. a Left: schematic representation of mouse *Unc5b* pre-mRNA encompassing exon 7 and 9. NOVA2 binding sites (YCAY sites) identified by RBPmap are shown as vertical bars. Bars of various colors indicate the different Z-scores calculated by RBPmap (orange: Z-score ≥ 3.50; pale orange: Z-score < 3.50 & ≥ 2.75; yellow: Z-score < 2.75 & ≥ 2.00). 100-nt length riboprobes for the *in vitro* pull-down assay are indicated below; in the mutated riboprobe, YCAY repeats were replaced with YAAY. *Wt* and *Mut* riboprobes contain at the 3' end TDP-43 binding motifs (CACACACACACACACAC; green segments). Right: *in vitro* RNA pull-down assay with NOVA2-HA over-expressing HeLa cellular extracts and the indicated riboprobes. Streptavidin resin only pull-down was loaded as an additional control (beads). TDP-43 immunoblotting as an internal control. **b** Left: silencing of RBFOX2 in moEC was verified by immunoblotting. VINCULIN as loading control. Center: RT-PCR analysis of *Unc5b* exon 8 splicing in the same ECs. Right: schematic representation of the RBFOX binding sites identified by using RBPmap. For RBPmap analysis, recognized motifs, statistical parameters (Z-score and P-value), and relative position to the *Unc5b* exon 7-intron 7 junction are also shown. The percentage of exon inclusion is shown below each gel. At least three independent biological replicates were analyzed for each experiment.

Supplementary Figure 5

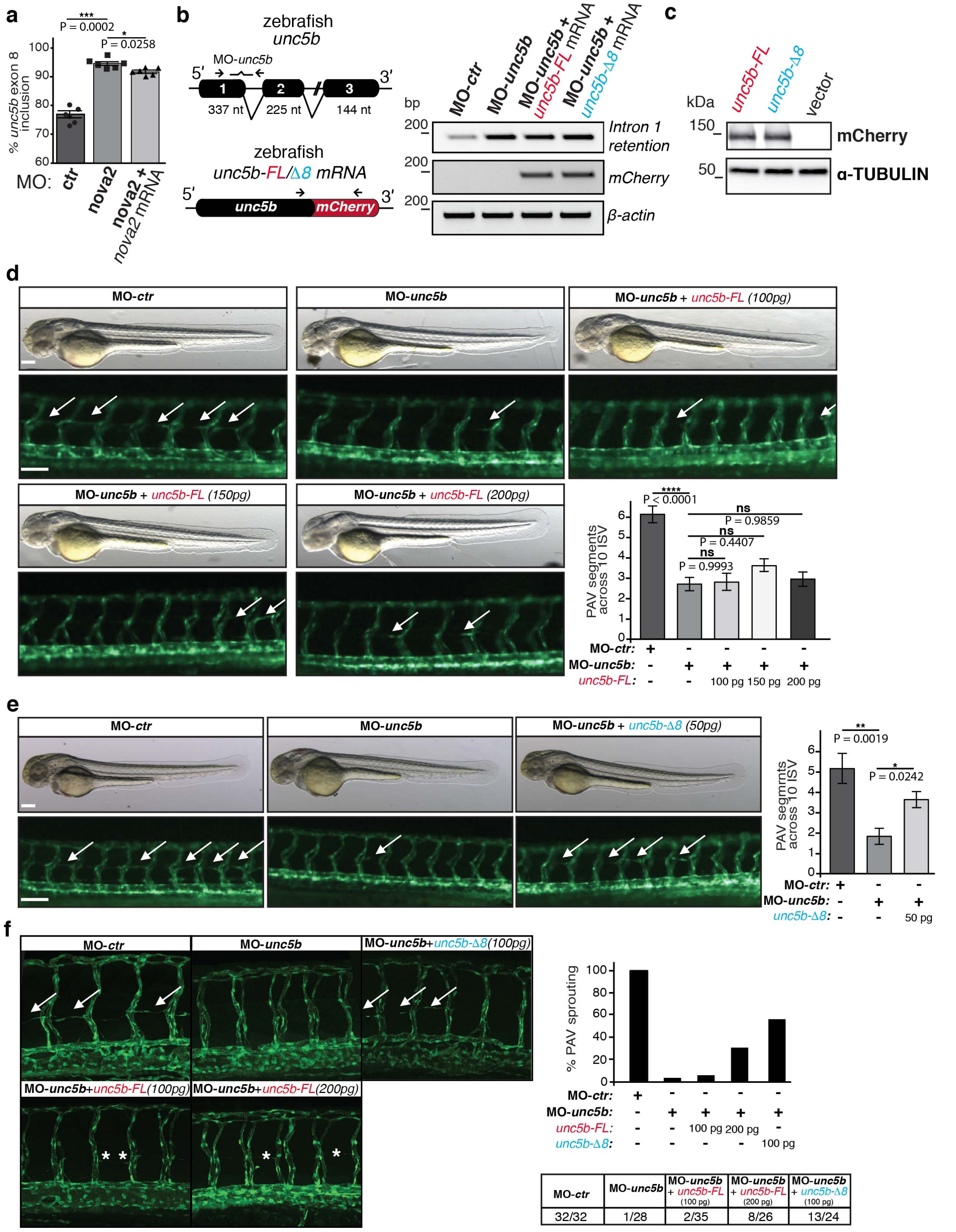

MO-*ctr*	MO-*unc5b*	MO-*unc5b* + *unc5b-FL* (100 pg)	MO-*unc5b* + *unc5b-FL* (200 pg)	MO-*unc5b* + *unc5b-Δ8* (100 pg)
32/32	1/28	2/35	8/26	13/24

Supplementary Fig. 5. Unc5b-Δ8 specifically promotes PAV formation in zebrafish. a Quantification of *unc5b* exon 8 inclusion (%) in zebrafish embryos (28 hpf) injected with a control morpholino (MO-*ctr*) or a morpholino against *nova2* (MO-*nova2*). *unc5b* exon 8 AS was partially corrected by the co-injection of a morpholino-resistant *nova2* mRNA (MO-*nova2*+*nova2* mRNA). Error bars = ±SEM. One-Way ANOVA for multiple comparisons test (n=6).

b Left: Schematic representation of the zebrafish *unc5b* genomic region encompassing exons 1, 2 and 3, and morpholino-resistant mCherry-tagged *unc5b-FL/Δ8* mRNAs. Black boxes = constitutive exons; lines = introns. *Unc5b* splice-blocking morpholino (MO-*unc5b*) annealing to *unc5b* exon1-intron 1 junction is also shown. Red box = sequence encoding the C-terminal mCherry tag. Black arrows indicate primers used for RT-PCR. Right: analysis of splicing/retention of *unc5b* intron 1 in zebrafish embryos (72 hpf) injected with a control morpholino (MO-*ctr*) or a morpholino against *unc5b* (MO-*unc5b*), and expression of co-injected morpholino-resistant *unc5b* mRNA variants (*unc5b-FL* and *unc5b-Δ8*). *β-actin* mRNA as internal control. **c** Immunoblotting with an anti-mCherry antibody of HeLa cells transiently transfected with expression vectors expressing zebrafish Unc5b-FL and Unc5b-Δ8, or the empty vector. α-TUBULIN as loading control. At least three independent biological replicates were analyzed. **d** Fluorescence and brightfield images of *Tg(kdrl:GFP)* ^{la116} *unc5b*-depleted zebrafish embryos (52 hpf) co-injected with different doses of *unc5b-FL* mRNA and quantification of PAV formation (right) (n=14,19,26,21,24). Arrow = PAV. Scale bar: 100 µm **e** Fluorescence and brightfield images of *Tg(kdrl:GFP)* ^{la116} *unc5b*-depleted zebrafish embryos (52 hpf) co-injected with a lower dose (50 pg per embryo) of *unc5b-Δ8* mRNA. Embryos injected with a control morpholino (MO-*ctr*) or (MO-*unc5b*) are also shown. Right: quantification of PAV segments across ten ISV is shown. (n=6,12,21). Arrow = PAV. Scale bar: 100 µm **f** Left: lateral views (fluorescence) of *Tg(kdrl:GFP)* ^{la116} zebrafish embryos (72 hpf) injected with a control morpholino (MO-*ctr*) or with a morpholino against *unc5b* (MO-*unc5b*); *unc5b*-morphants were also co-injected with two different doses of *unc5b-FL* mRNA (100 pg or 200 pg per embryo) or *unc5b-Δ8* mRNA (100 pg per embryo). Asterisks indicate initiation of PAV sprouting. Arrow = PAV. Right: quantification of PAV in which sprouting initiation was observed upon co-injection of *unc5b-FL* and *unc5b-Δ8* mRNA at different doses. One-way ANOVA for multiple comparisons. Error bars indicate ±SEM. Exact P-values (P) are indicated: * < 0.05; ** < 0.01; *** < 0.001; **** < 0.0001. ns = not significant.

Supplementary Figure 6

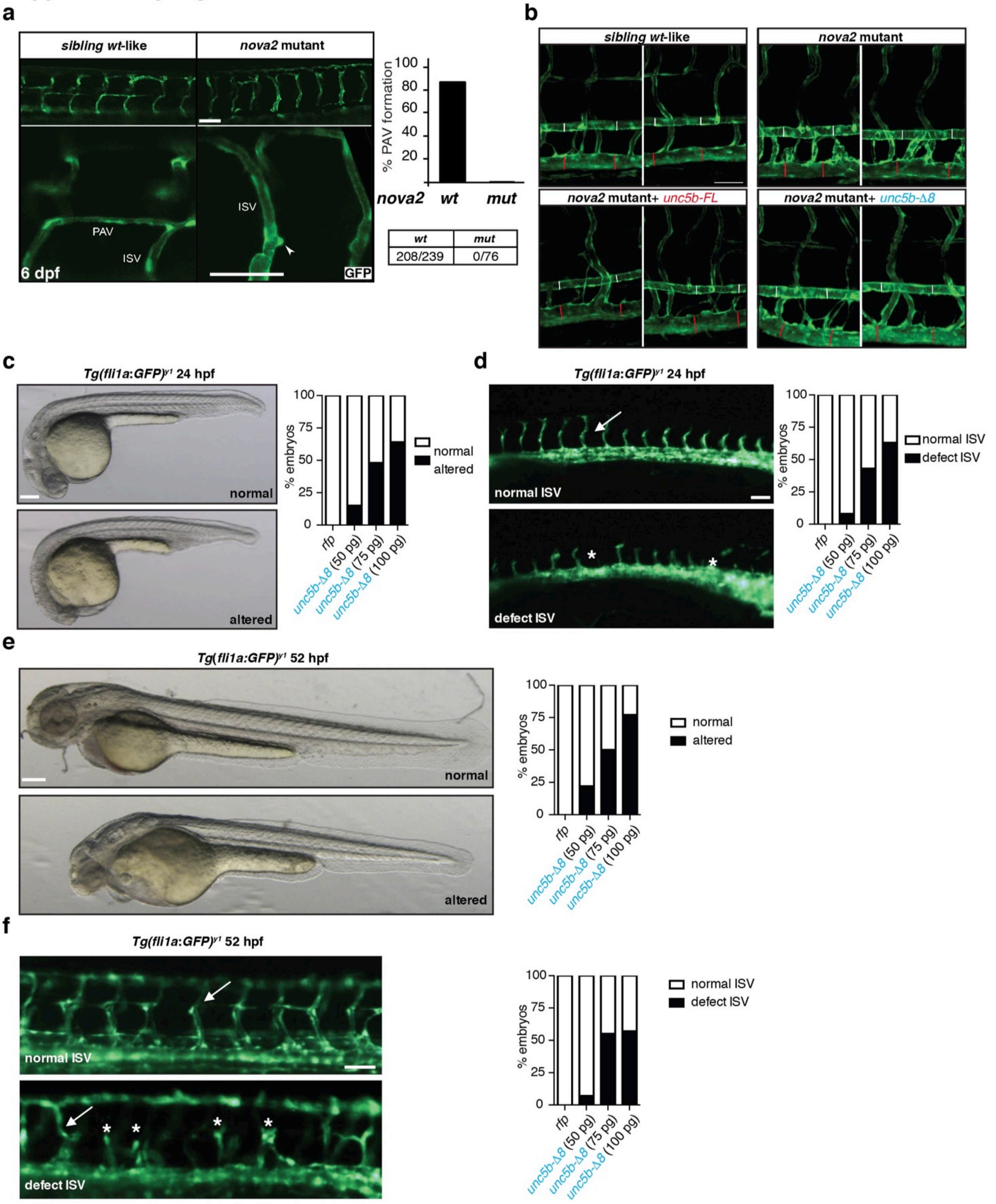

Supplementary Fig. 6. Unc5b-Δ8 overexpression characterization in a wild-type background. a Left: lateral views (fluorescence) of 6 dpf *Tg(kdrl:GFP)* *la116* sibling wild-type like and *nova2* CRISPR mutant. Right: quantification of PAV formation in 6 dpf wild-type (*wt*) and *nova2* CRISPR mutants (*mut*). N. of larvae with normal PAV out the total n. of larvae. Scale bar: 100 µm. **b** Lateral views (fluorescence) of 5 dpf sibling *wt*-like, *nova2* mutant, and *nova2* mutant injected with *unc5b* mRNA variants (*unc5b-FL* and *unc5b-Δ8*). Dorsal aorta (DA; white) and posterior cardinal vein (PCV, red) diameters are highlighted. Scale bar: 25 µm. **c** Morphological analysis of 24 hpf *Tg*(*fli1a:GFP*)y1 embryos

injected with different doses of *unc5b-Δ8* mRNA. The percentage of embryos with an altered phenotype is reported in the graph on the right. Scale bar: 100 μm **d** Evaluation of intersegmental vessel (ISV) defects in the above embryos. Arrow indicates a correctly formed ISV. Asterisks indicate defective ISVs. Scale bar: 100 μm **e** and **f** Morphological and ISV formation analyses of *Tg(fli1a:GFP)^{y1}* embryos injected with different doses of *unc5b*-Δ8 mRNA are also shown at 52 hpf. Scale bar: 100 μm.

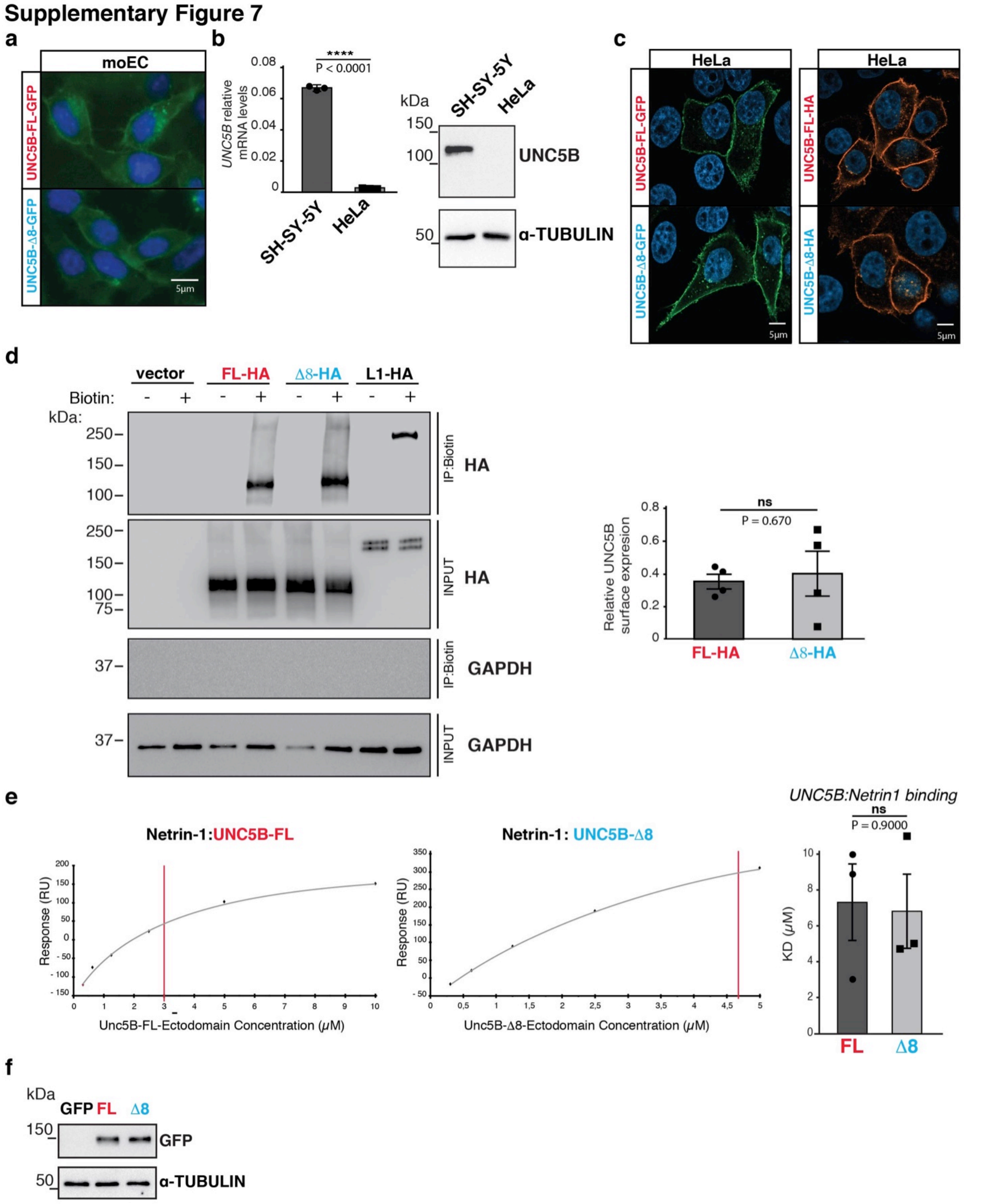

Supplementary Fig. 7. Localization and Netrin-1 binding of UNC5B isoforms. a Membrane localization of UNC5B-FL and UNC5B-Δ8 in inducible moEC. Scale bar is indicated. **b** Analysis by RT-qPCR (relative to *ACTB*) and immunoblotting of UNC5B expression levels in HeLa and, as positive control, in SH-SY-5Y cells. α-TUBULIN as loading control. Two-tailed Student's t-test. Error bars indicate ±SEM. Exact P-value (P) is indicated: **** < 0.0001; **c** Membrane localization of GFP-tagged and HA-tagged UNC5B isoforms in transiently transfected HeLa cells. Scale bars are indicated. At least three independent biological replicates were analyzed. **d** Left: pull-down of surface

biotinylated membrane proteins in HeLa cells transiently transfected with HA-tagged UNC5B isoforms, the L1-CAM transmembrane receptor (L1) as positive control, or with the empty vector (vector). GAPDH as internal control. Right: quantification of UNC5B isoforms relative surface expression (IP/INPUT ratio). n=4. Two-tailed Paired Student's t-test. Exact P-value (P) is indicated: ns = not significant. **e** Representative surface plasma resonance (SPR) analyses of recombinant UNC5B-FL (left) and UNC5B-Δ8 ectodomains (center) binding with Netrin-1 on a BIAcore T200. Red bars indicate the equilibrium dissociation constant (KD). KD was calculated for three independent experiments for each ectodomain (right). **f** Immunoblotting (anti-GFP) of UNC5B AS isoforms in transiently transfected HeLa cells with plasmids encoding either UNC5B-FL-GFP, UNC5B-Δ8-GFP, or GFP. α-TUBULIN as loading control. At least three independent biological replicates were analyzed.

Supplementary Figure 8

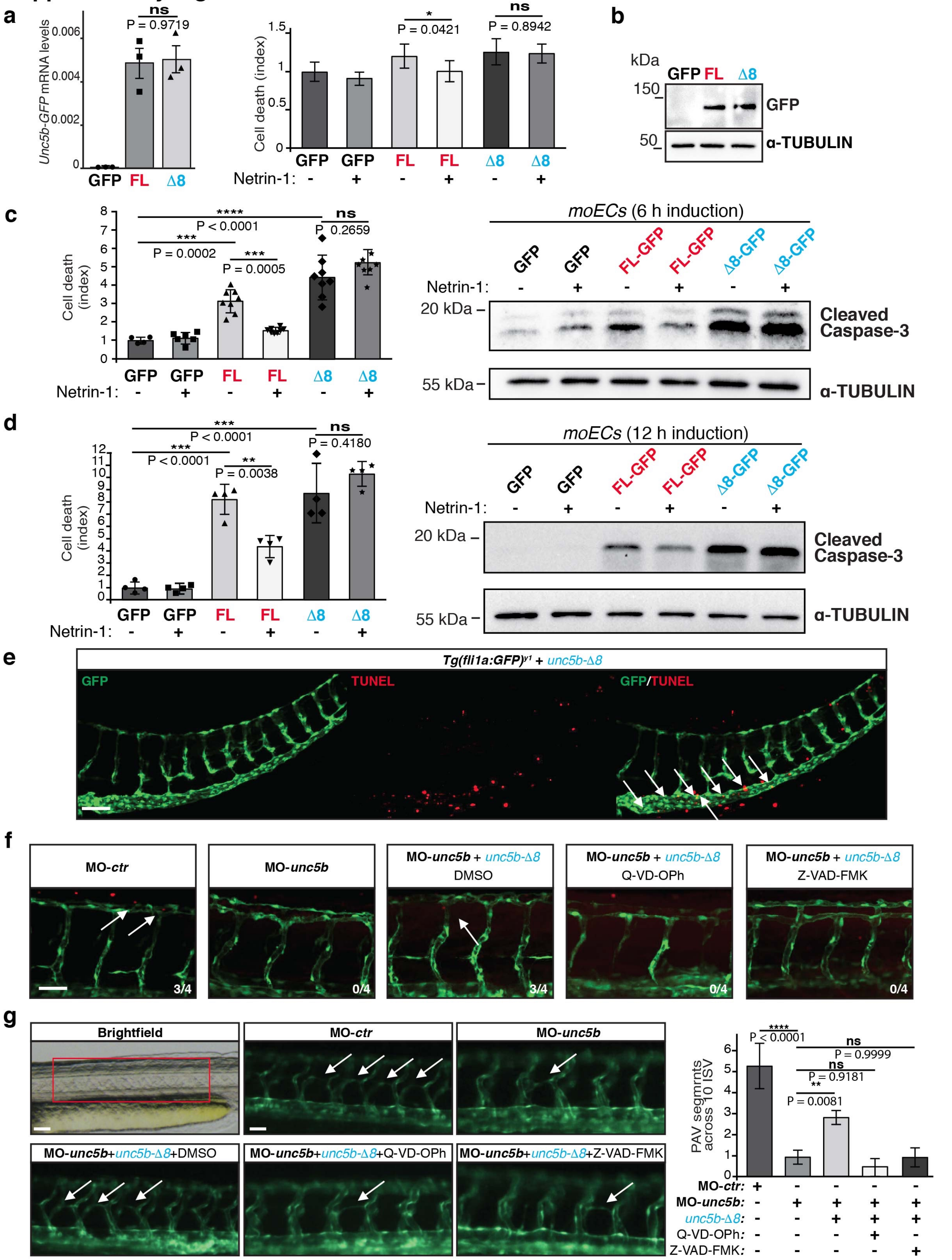

Supplementary Fig. 8. UNC5B-Δ8 prevents Netrin-1 pro-survival signaling in ECs. a Left: analysis by RT-qPCR (relative to *GAPDH*) of *UNC5B*-AS isoforms (FL and Δ8) transduced in HUVEC. Right: Caspase-3 activation in HUVEC transduced with adenoviral vectors expressing UNC5B-FL-GFP, UNC5B-Δ8-GFP, or GFP treated with or without Netrin-1 (150 ng/ml). n=3. **b** Immunoblotting (anti-GFP) of UNC5B AS isoforms in moEC transduced with

inducible UNC5B-FL-GFP, UNC5B-Δ8-GFP or GFP induced with doxycycline for 12 h. α-TUBULIN as loading control. Two-tailed Paired Student's t-test. n=12; error bars indicate ±SEM. Exact P-value (P) is indicated: * < 0.05; ns: not significant. **c** Vital exclusion assay (left) and immunoblotting against the cleaved (activated form) of Caspase-3 (right) in the above moEC induced with doxycycline for 6 hr (n = 4,6,8,8,8,8) or (**d**) 12 hr in the presence of Netrin-1 (150 ng/ml) (n=4); α-TUBULIN as loading control. One-way ANOVA for multiple comparison; error bars indicate ±SD. Scale bar: 100 µm. **e** Cell death evaluation, by TUNEL assay, on 48 hpf *Tg(fli1a:GFP)^{y1}* wild-type zebrafish embryos injected with 100 pg (per embryo) of *unc5b-Δ8* mRNA. Dying ECs are indicated by arrows. **f** TUNEL assay in 52 hpf *Tg(kdrl:GFP)^{la116}* injected with a control morpholino (MO-*ctr*) or with a morpholino against *unc5b* (MO-*unc5b*); *unc5b*-morphants were also co-injected with 50 pg of a morpholino-resistant zebrafish *unc5b-Δ8* and treated with two different caspase inhibitors (Q-VD-Oph and Z-VAD-FMK). Arrows indicate TUNEL-positive ECs. For each condition the number of embryos with co-localization of GFP and TUNEL signals is provided. Scale bar: 100 µm. **g** Lateral views (fluorescence) of the above zebrafish embryos. Arrows: forming PAV. The analyzed region of zebrafish trunk is highlighted in the red box in the brightfield Right: Quantification of PAV formation in the above zebrafish embryos. n= 6,17,9,12,12. Scale bar: 100 µm. One-way ANOVA for multiple comparison; error bars indicate ±SEM. Exact P-value (P) is indicated: ** < 0.01; *** < 0.001; **** < 0.0001; ns: not significant.

Supplementary Figure 9

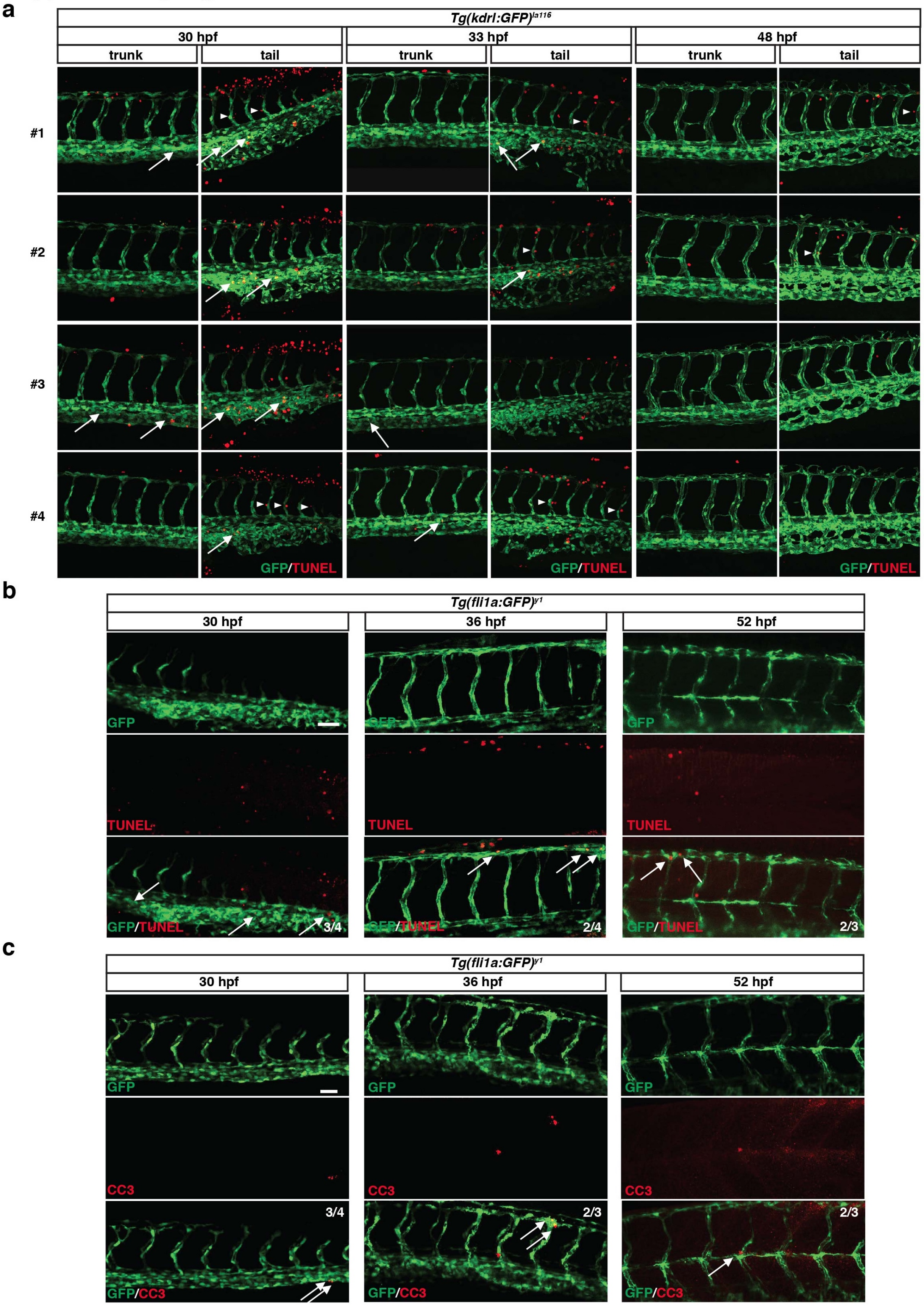

Supplementary Fig. 9. EC apoptosis during zebrafish development. a Terminal deoxynucleotidyl transferase (TdT) dUTP Nick-End Labeling (TUNEL) assay in *Tg(kdrl:GFP)* *la116* zebrafish embryos at 30 hpf, 33 hpf, and 48 hpf developmental stages. The trunk and tail regions of four different embryos (#1-4) are shown. Arrows indicate TUNEL-

positive ECs. Arrowheads indicate dying cells in close proximity to PAV sprouting region in the ISV. **b** Presence of TUNEL-positive ECs was also confirmed in *Tg(fli1a:GFP)*^{y1} embryos at 30 hpf; 33 hpf, and 52 hpf. Arrows indicate TUNEL-positive ECs. The number of embryos with co-localization of GFP and TUNEL signals is provided. Scale bar: 100 µm. **c** Apoptotic cell-death in the above *Tg(fli1a:GFP)*^{y1} zebrafish embryos was confirmed by immunostaining for cleaved Caspase-3 (CC3) immunostaining. Co-localization of EC-restricted GFP and CC3 signals are indicated by arrows. The number of embryos with co-localization of GFP and CC3 signals is provided. Scale bar: 100 µm.

Supplementary Figure 10

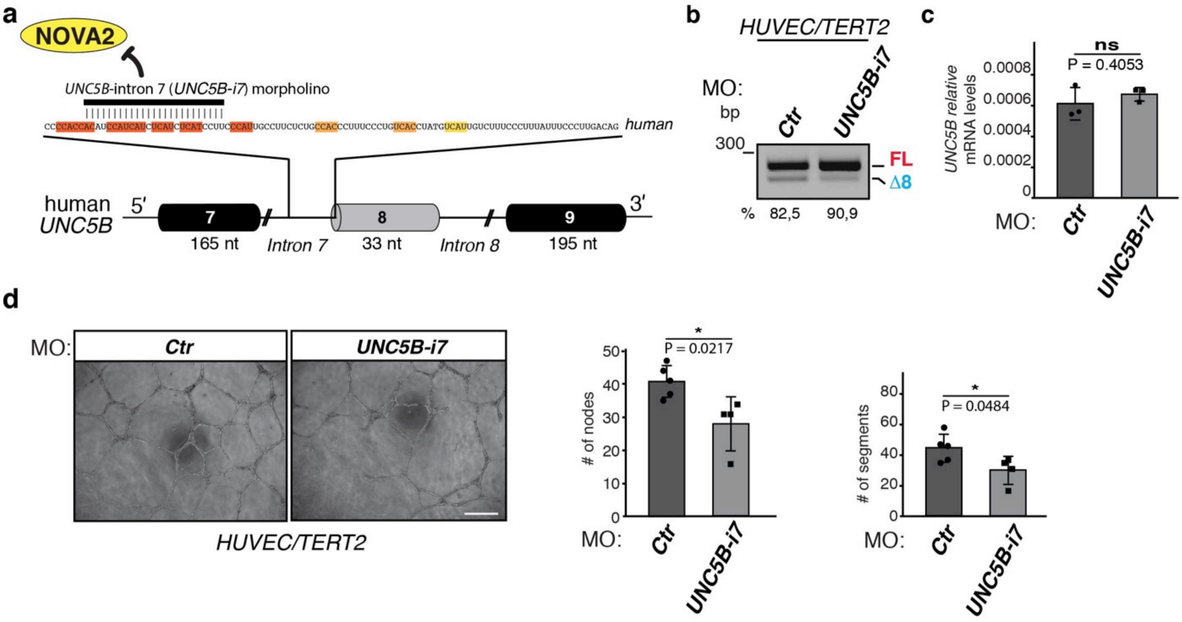

Supplementary Fig. 10. Morpholino-mediated inhibition of *UNC5B-Δ8* production reduces tube formation capabilities of human ECs. a Schematic representation of the human *UNC5B* pre-mRNA with the NOVA2 binding sites (YCAY cluster predicted by RBPmap tool and indicated in different colors according to their calculated Z-score) and the complementary annealing region of the morpholino oligo (*UNC5B-i7*) in intron 7. **b** RT-PCR analysis of *UNC5B* exon 8 splicing in HUVEC/TERT2 treated with *UNC5B-i7* or a control morpholino oligo (*Ctr*). The percentage of exon 8 inclusion is shown below the gel. **c** RT-qPCR (relative to *ACTB*) of total *UNC5B* expression levels in the same HUVEC/TERT2 cells. Two-tailed Student's t-test; error bars indicate ±SEM. Exact P-value (P) is indicated: ns = not significant. n=3. **d** Left: *in vitro* angiogenesis assay of HUVEC/TERT2 treated with *UNC5B-i7* morpholino or a MO-*Ctr* morpholino oligos and plated on Matrigel-coated culture plates. Scale bar: 0.5 mm. Right: quantification of the number (#) of nodes and segments. One experiment of three different biological replicate is shown (n=5,4). Two-tailed Student's t-test; error bars indicate ±SD. Exact P-values (P) are indicated: * < 0.05; ns = not significant.

Supplementary Figure 11

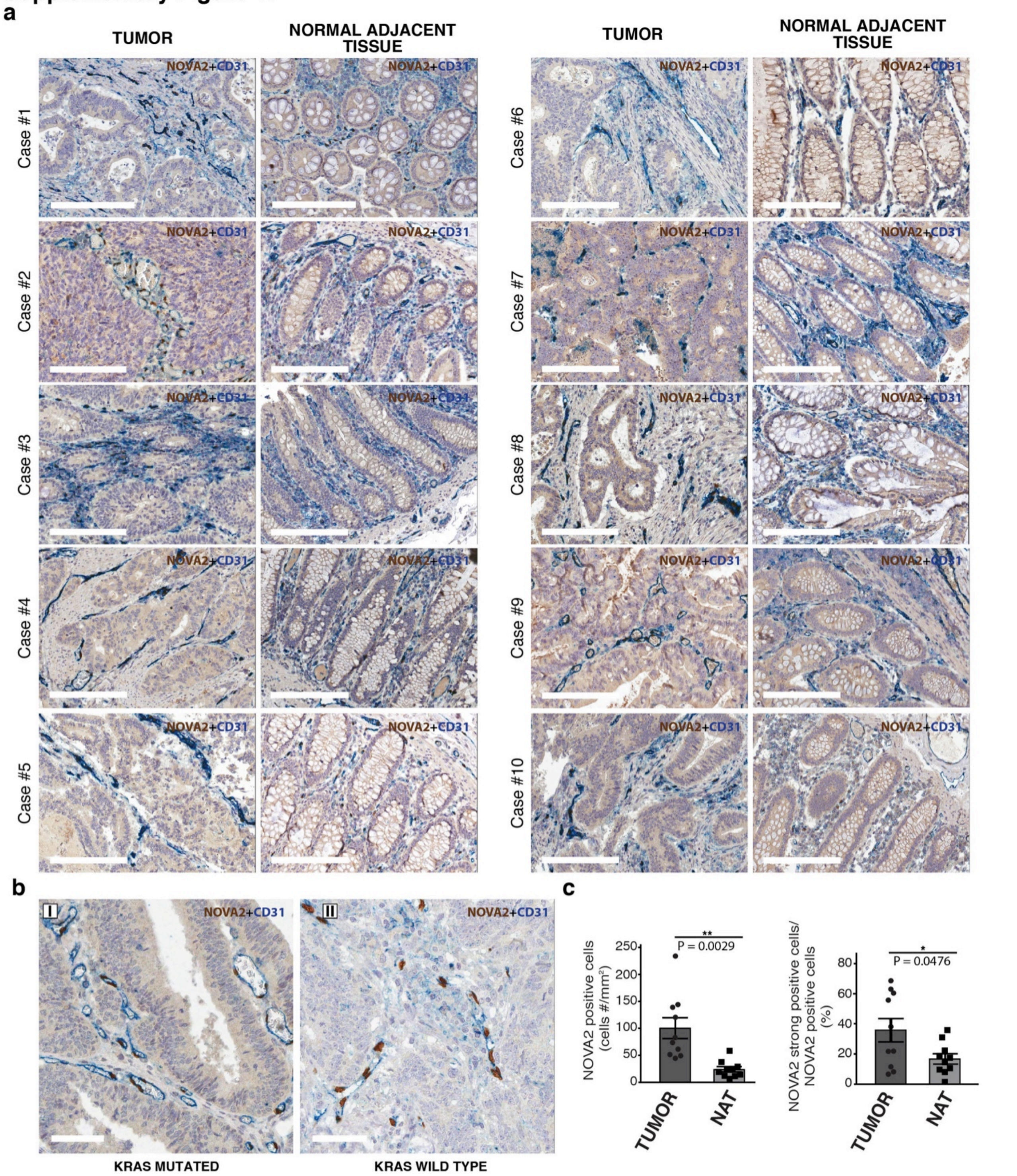

Supplementary Fig. 11. NOVA2 is upregulated in ECs of human colon cancer vasculature. a IHC images for double staining of NOVA2 and the vessel marker CD31 in 10 cases of colorectal cancer patients. For each case tumor and normal adjacent tissue are shown. Scale bars: 200 µm. **b** Representative IHC images for double staining of NOVA2 and CD31 in KRAS mutated (I) or wild type (II) colon cancer samples (n = 4). NOVA2 is selectively expressed by tumor ECs co-expressing CD31. Scale bars: 60 µm, magnification 400X. **c** Quantification of NOVA2 positive cells (left) and NOVA2 positive cells with strong IHC signal (right) relative to total cell area and total NOVA2 positive cells, respectively, in tumor specimens (TUMOR) and normal adjacent tissue (NAT). Two-tailed Student's t-test with Welch's correction. Error bars indicate ±SEM. Exact P-values (P) are indicated: * P-value < 0.05; ** P-value < 0.01.

Supplementary Figure 12

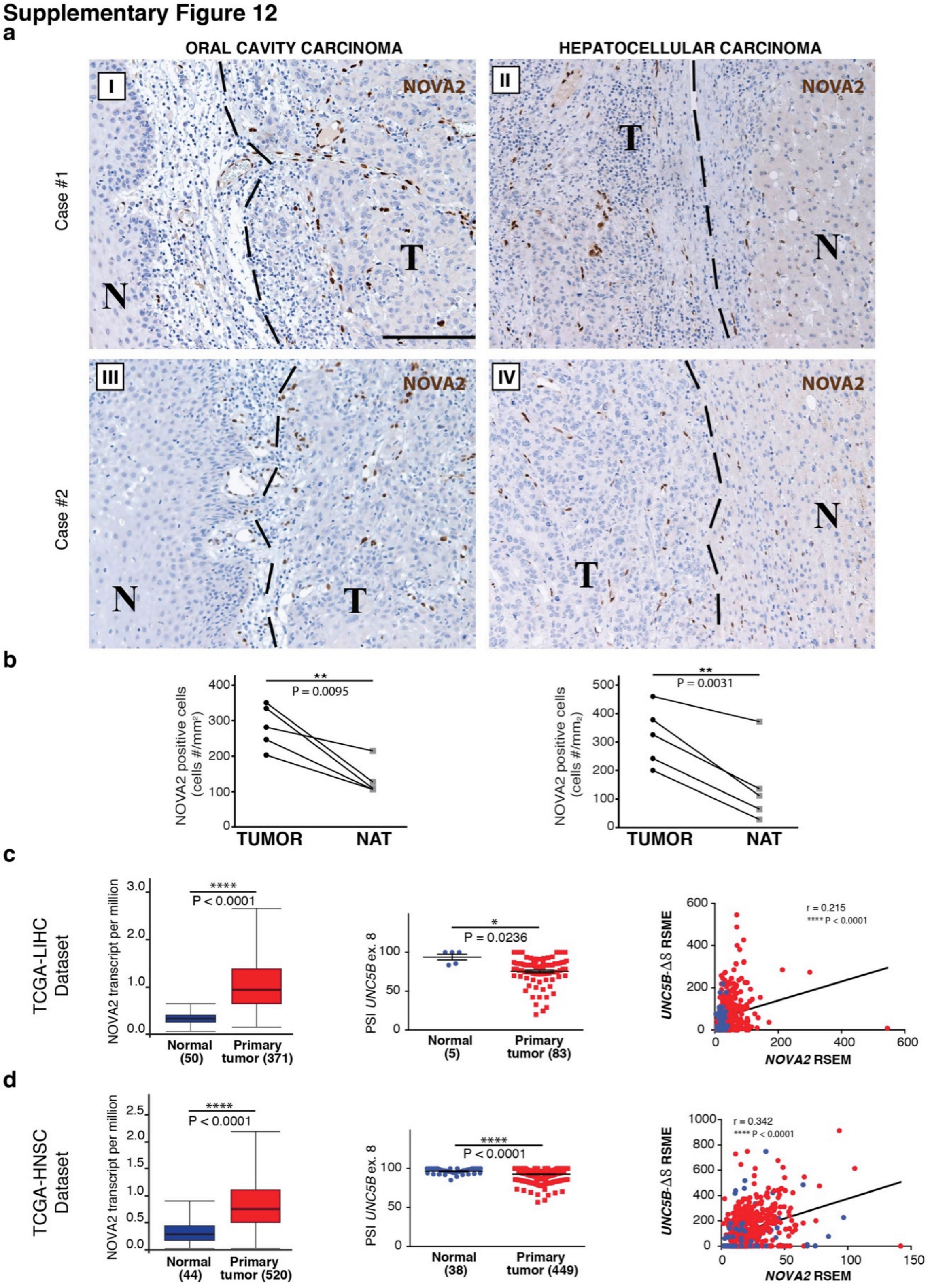

Supplementary Fig. 12. NOVA2 is upregulated in ECs of different human carcinomas. a Representative IHC images for NOVA2 staining in two cases of oral cavity carcinoma (I and III) and in two cases of hepatocarcinoma (II and IV). NOVA2 shows upregulation in tumor ECs. Normal adjacent tissue (N) and tumor (T) areas are shown. Scale bars: 200 µm. n=5. **b** Quantification of NOVA2 positive cells relative to total cells are in tumor specimens (TUMOR) and normal adjacent tissue (NAT). Two-tailed paired Student's t-test. Error bars indicate ±SEM. Exact P-values (P) are indicated: ** < 0.01. **c** RNA-seq data analysis of *NOVA2* mRNA expression levels, PSI (Percent Spliced-In) of *UNC5B*

exon 8, and correlation between *NOVA2* and *UNC5B-Δ8* expression in normal (blue) and primary tumor (red) samples in the TCGA-LIHC (Liver Hepatocellular Carcinoma) and (**d**) TCGA-HNSC (Head-Neck Squamous Cell Carcinoma) datasets, respectively. Left: Box plot represents upper quartile, median, lower quartile. Error bars indicate minimum and maximum. Two-tailed Log-ranked test. Exact P-values (P) are indicated: **** < 0.0001. Centre: Two-tailed paired Student's t-test. Error bars indicate ±SEM. Exact P-values (P) are indicated: * P-value < 0.01; **** < 0.0001. Right: For correlation analyses Pearson coefficients (r), associated P values, and regression lines are shown.

Supplementary Figure 13

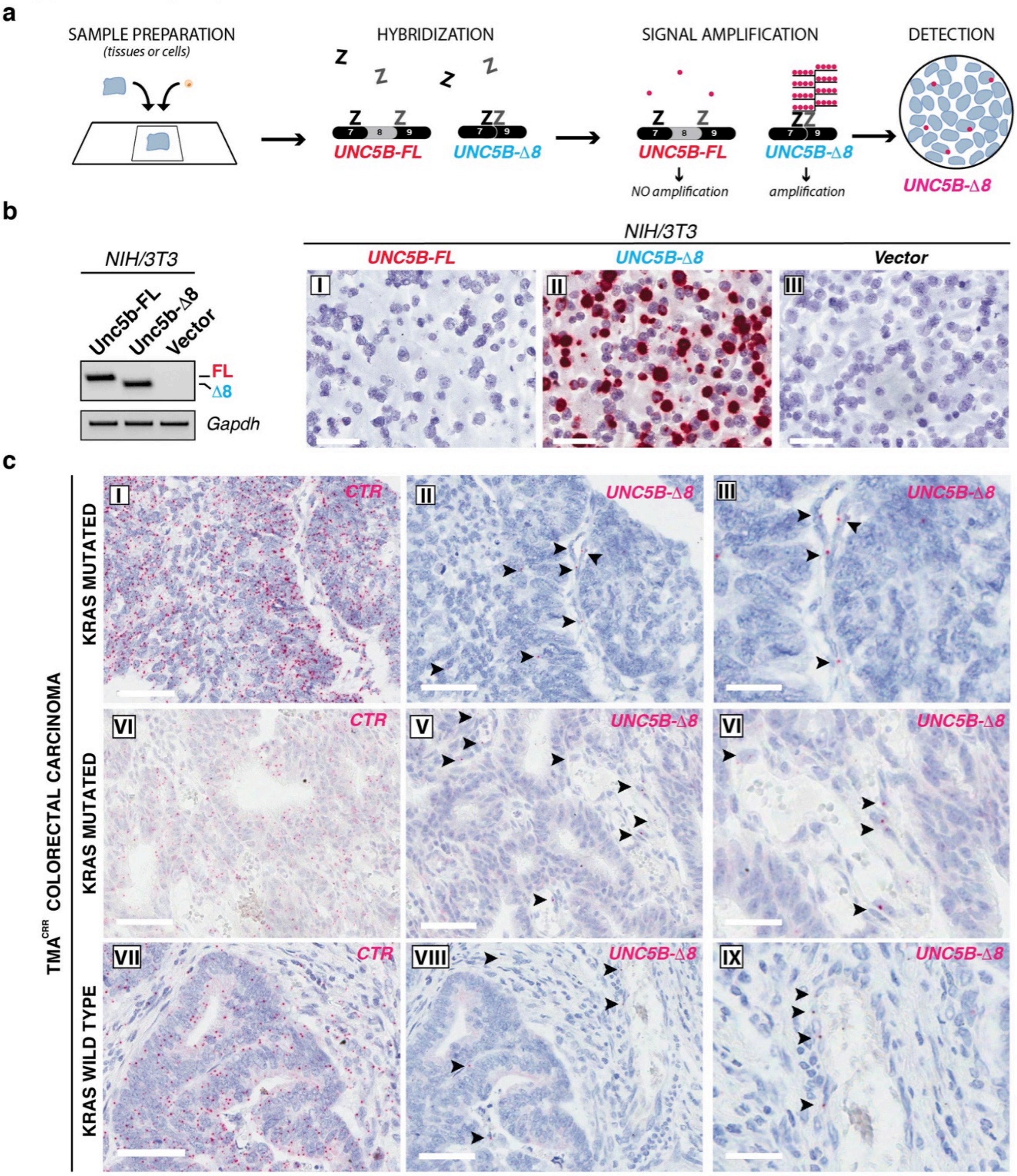

Supplementary Fig. 13. *UNC5B-Δ8* chromogenic BaseScope probe validation and *UNC5B-Δ8* selectively expression by colon cancer ECs. a Schematic representation of the ISH assay with a chromogenic BaseScope probe (Z) specific for *UNC5B-Δ8* mRNA. Signal amplification occurs only in *UNC5B* transcripts deleted of exon 8 in which a single pair of BaseScopes probes (ZZ) is constituted. Visualization of the *UNC5B-Δ8* mRNA expression is indicated as red dots. **b** Right: RT-PCR analysis of NIH/3T3 mouse fibroblasts transfected with human *UNC5B-FL*, *UNC5B-Δ8* cDNA, or the empty vectors. *Gapdh* as internal control. Left: the same cells were also analyzed by ISH with the *UNC5B-Δ8* probe. No signal was observed in NIH/3T3 cells expressing *UNC5B-FL.* **c** Representative images of ISH in the colon cancer samples with KRAS mutated (I-IV) or wild type (VII-IX) by using the positive CTR or the *UNC5B-Δ8* BaseScope probes. Whereas the signal from the CTR probe is widespread in different tumor cell types (I, IV, VII), *UNC5B-Δ8* expression is restricted to ECs (II, V, VIII), as highlighted in the corresponding high-power view (III, VI, IX). n=4. I, II, IV, V, VII, VIII scale bars: 60 µm, magnification: 400X; III, VI, IX scale bars 30 µm, magnification 800X.

Supplementary Figure 14

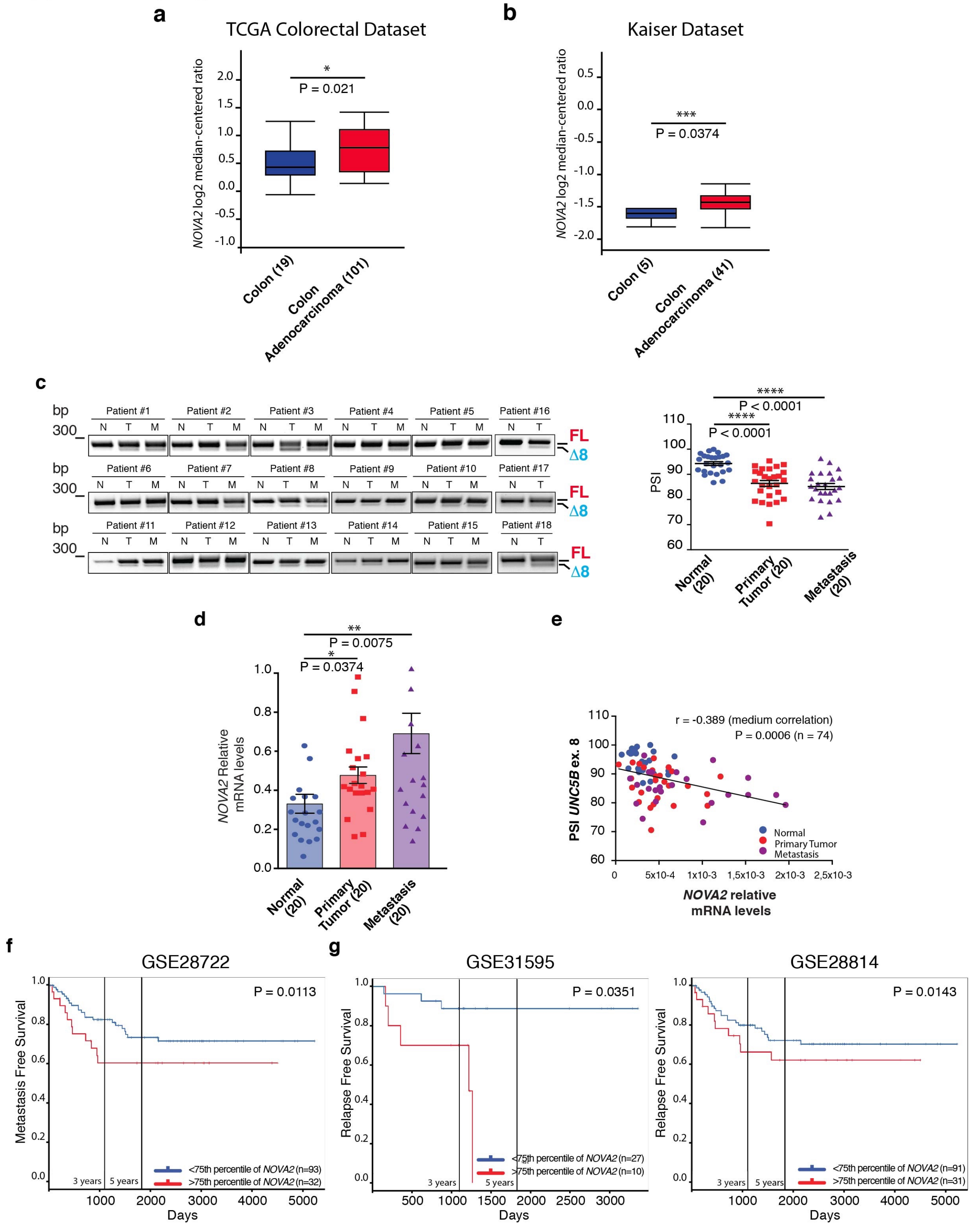

Supplementary Fig. 14. NOVA2/UNC5B-Δ8 axis in colon cancer patients. a *NOVA2* mRNA expression levels (microarray analysis) from *Oncomine* (http://oncomine.org/resource) in TCGA Colorectal and (**b**) Kaiser datasets. Box plot represents upper quartile, median, lower quartile. Error bars indicate minimum and maximum. **c** *UNC5B* splicing analysis by RT-PCR in normal colon tissues [N], colon primary tumors [T] and liver metastasis [M]. Quantification of PSI (Percent Spliced-In) of *UNC5B* exon 8 on the right. **d** *NOVA2* mRNA levels by RT-qPCR (relative to *ACTB*) in the

above normal, primary tumor, and metastasis specimens. RM one-way ANOVA; Error bars ±SEM. **e** Correlation between *NOVA2* expression and *UNC5B* splicing (PSI) in normal colon tissue (blue circle), colon primary tumor (red circle) and liver metastasis (violet circle). Linear regression (black line) and Pearson r coefficient with two-tailed P value are also calculated. **f** Kaplan-Meier plot of metastasis-free (GSE28722) and (**g**) relapse-free survival in colon adenocarcinoma patients classified according to *NOVA2* expression (GSE31595 and GSE28814). Blue curve: low expression (< 75th percentile); red curve: high expression (>75th percentile); data from PROGgeneV2 tool (http://genomics.jefferson.edu/proggene). Two-tailed Student's t-test. Exact P-values (P) are indicated: * < 0.05; ** < 0.01; **** < 0.0001.

Supplementary Figure 15

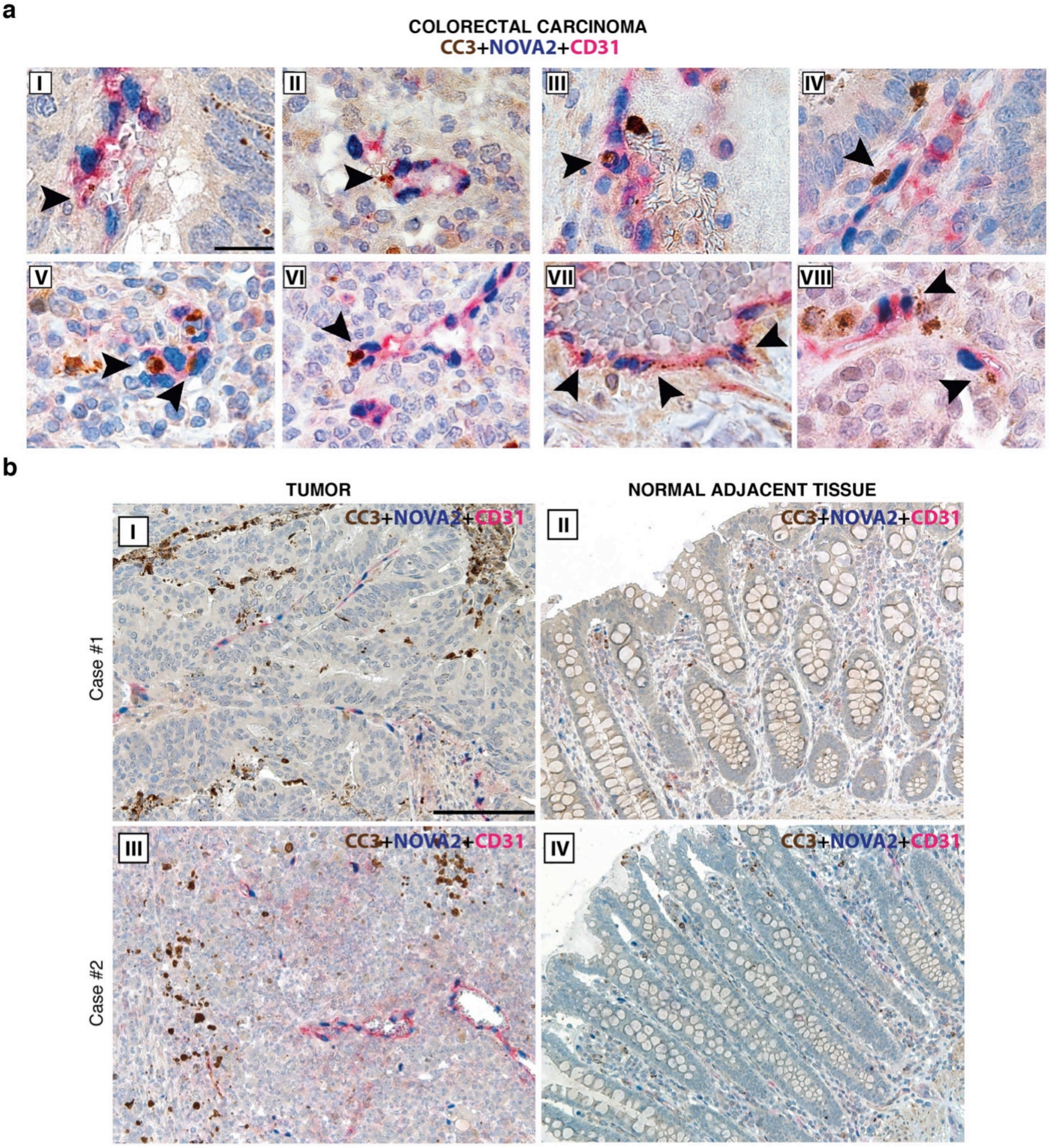

Supplementary Fig. 15. Apoptosis in NOVA2-positive human colon cancer ECs. a Representative IHC images for triple staining of NOVA2, the endothelial marker CD31, and cleaved Caspase-3 (CC3) in human colorectal cancer specimens. Black arrowheads indicate apoptotic (cleaved Caspase-3 positive) tumor ECs (CD31 positive) that shown also NOVA2 nuclear signal. n=8. Scale bar: 30 µm. **b** Representative IHC images for triple staining of NOVA2, the endothelial marker CD31, and cleaved Caspase-3 (CC3) in tumor (I and III) and normal adjacent tissue (II and IV) colorectal specimens. n = 4. Scale bar: 200 µm.

Supplementary Figure 16

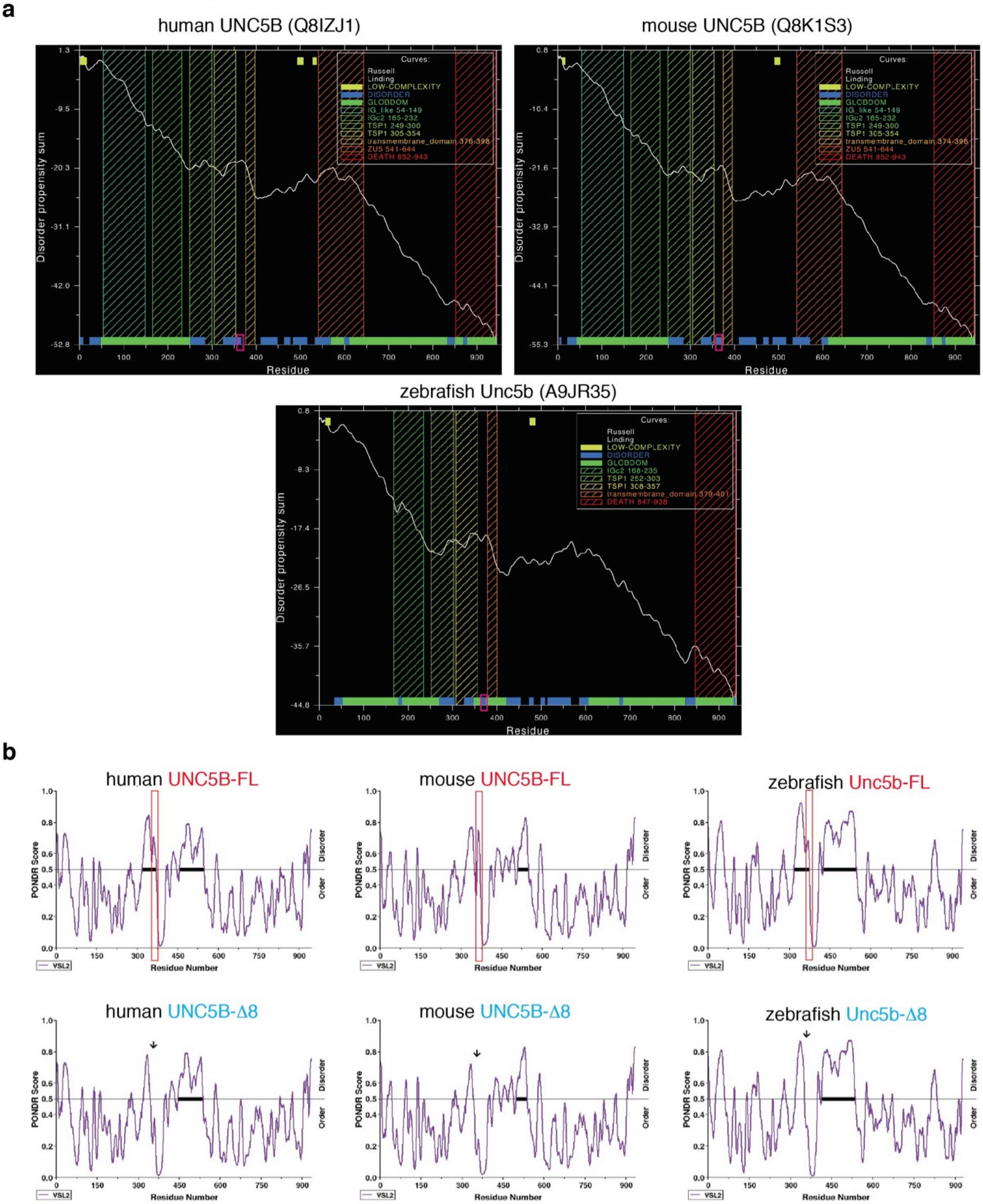

Supplementary Fig. 16. Identification of UNC5B intrinsically disordered region (IDR). a GlobPlot prediction analysis (http://globplot.embl.de) of human (Q8IZJ1), mouse (Q8K1S3), and zebrafish (A9JR35) UNC5B proteins. Low-complexity (yellow), disordered (blue), and globular (green) regions are shown on the x-axis, where UNC5B residues are indicated. Violet box indicates the position of the 11 residues encoded by exon 8. Predicted UNC5B domains and their relative position are shown in different colors: IG-like domain (aqua green), Igc2 domain (dark green), TSP1 (light green); TSP1 (yellow), transmembrane domain (pale orange); ZU5 domain (orange); death domain (red). Russell-Linding cumulative propensity of disorder is also shown (white line). **b** Graphic representation of PONDR prediction output (http://www.pondr.com), obtained by using the VLS2 algorithm optimized for long and short

disordered regions, of human, mouse, and zebrafish UNC5B-FL (upper panels) and UNC5B-Δ8 (lower panels) protein isoforms. PONDR score for each residue is shown. Red boxes indicate the position of the 11 residues encoded by exon 8 in UNC5B-FL. Black arrows indicate the absence of a disorder peak (PONDR score > 0.5) in UNC5B-Δ8 region where exon 8 is missing.

Supplementary Figure 17

a

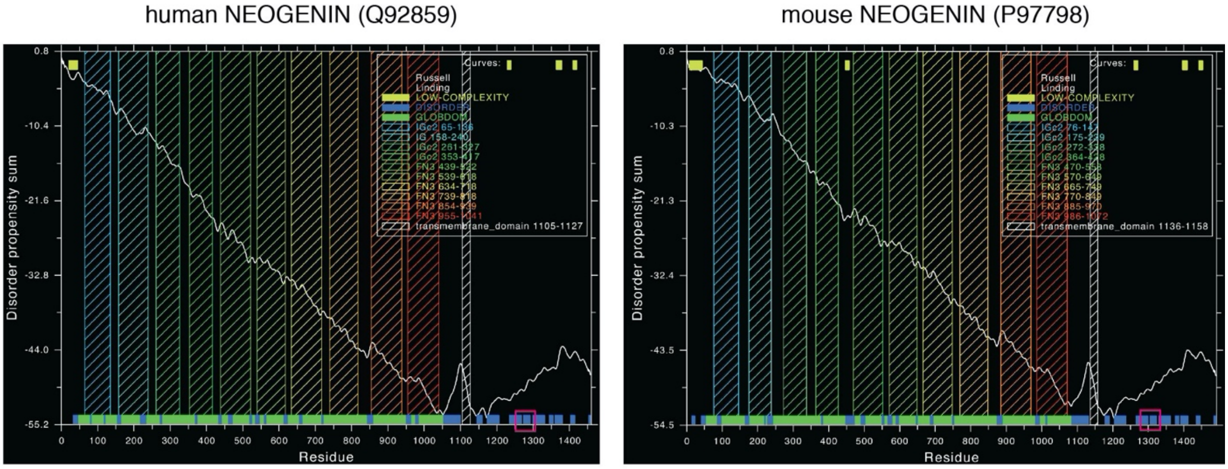

Supplementary Fig. 17. Identification of NEOGENIN intrinsically disordered region (IDR). a GlobPlot prediction analysis (http://globplot.embl.de) of human (Q92859) and mouse (P97798) NEOGENIN proteins. Low-complexity (yellow), disordered (blue), and globular (green) regions are shown on the x-axis, where NEOGENIN residues are indicated. Violet box indicates the position of the 53 residues encoded by exon 27. Predicted NEOGNIN domains and their relative position are shown in different colors: Igc2 domain (light blue, azure, aqua green, and green), FN3 (light green, yellow, pale orange, orange, and red), transmembrane domain (white). Russell-Linding cumulative propensity of disorder is also shown (white line).

Supplementary Figure 18

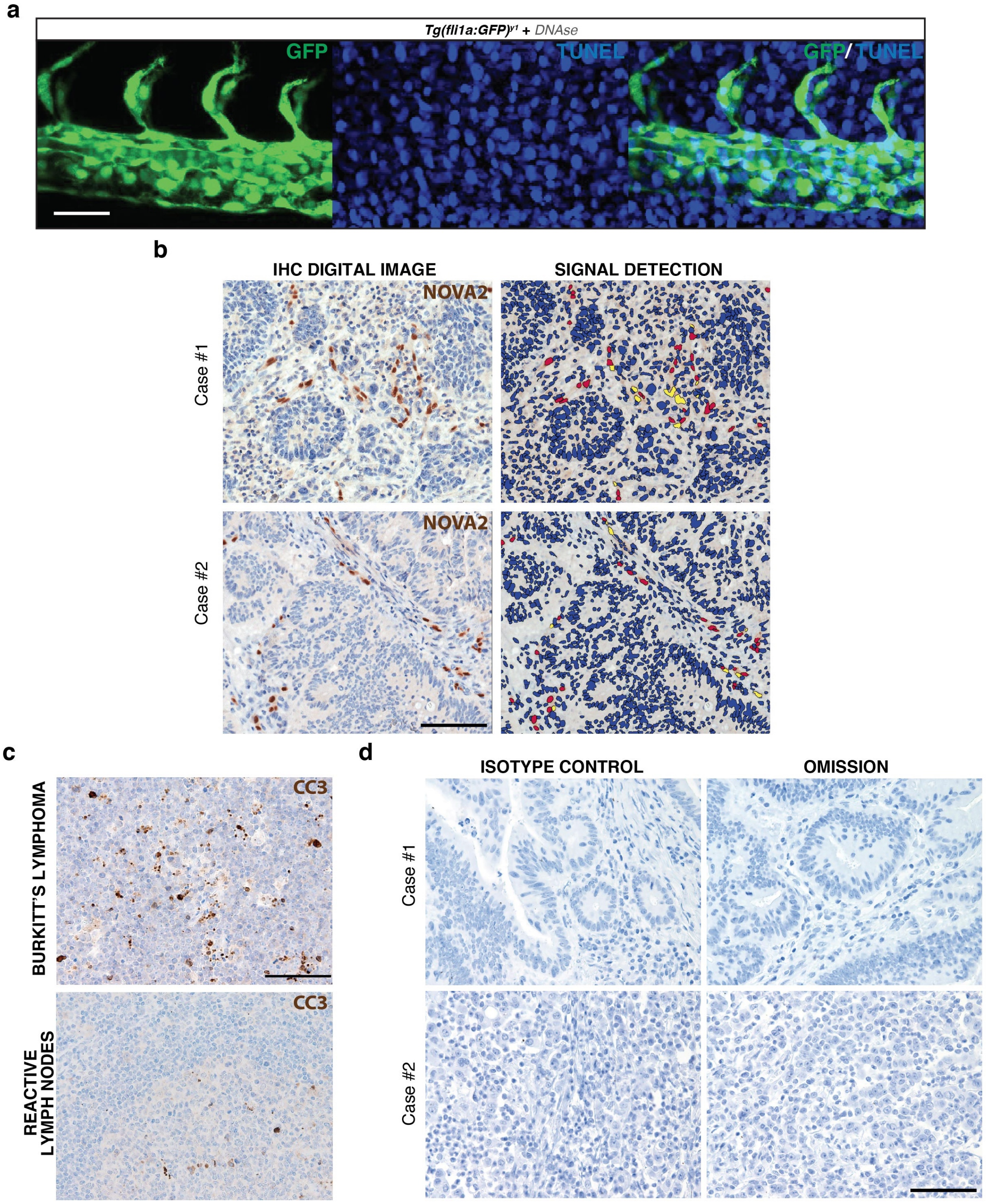

Supplementary Fig. 18. Validation of TUNEL analysis in zebrafish embryo, representative images for NOVA2^{+} ECs quantification in human tumor specimens, and validation of cleaved Caspase-3 IHC analysis. a Representative images of 24 hpf *Tg(kdrl:GFP)* ^{la116} zebrafish embryos treated with DNAse and stained with TUNEL (terminal deoxynucleotidyl transferase dUTP nick end labeling). GFP signal, TUNEL signal, and composite image are shown. Scale bar: 100 µm. **b** On the left column native images are from two representative human CRC specimens stained for NOVA2 and counterstained with hematoxylin. On the right column, the corresponding processed areas are shown. Strongly positive cells (red) and weakly positive cells (yellow) are illustrated in the right column. Images acquired from digital slides using 200X magnification have been digitally resized using Adobe Photoshop. Scale bar: 200 µm. **c** Burkitt's lymphoma as Cleaved Caspase-3 (CC3) staining positive control. Reactive lymph nodes as a negative control. Scale bar: 200 µm. **d** Isotype and omission controls of Cleaved Caspase-3 (CC3) antibody in two cases (Case #1 and #2) of human CRC counterstained with hematoxylin. Scale bar: 200 µm.

Supplementary Figure 19. Uncropped original blots.

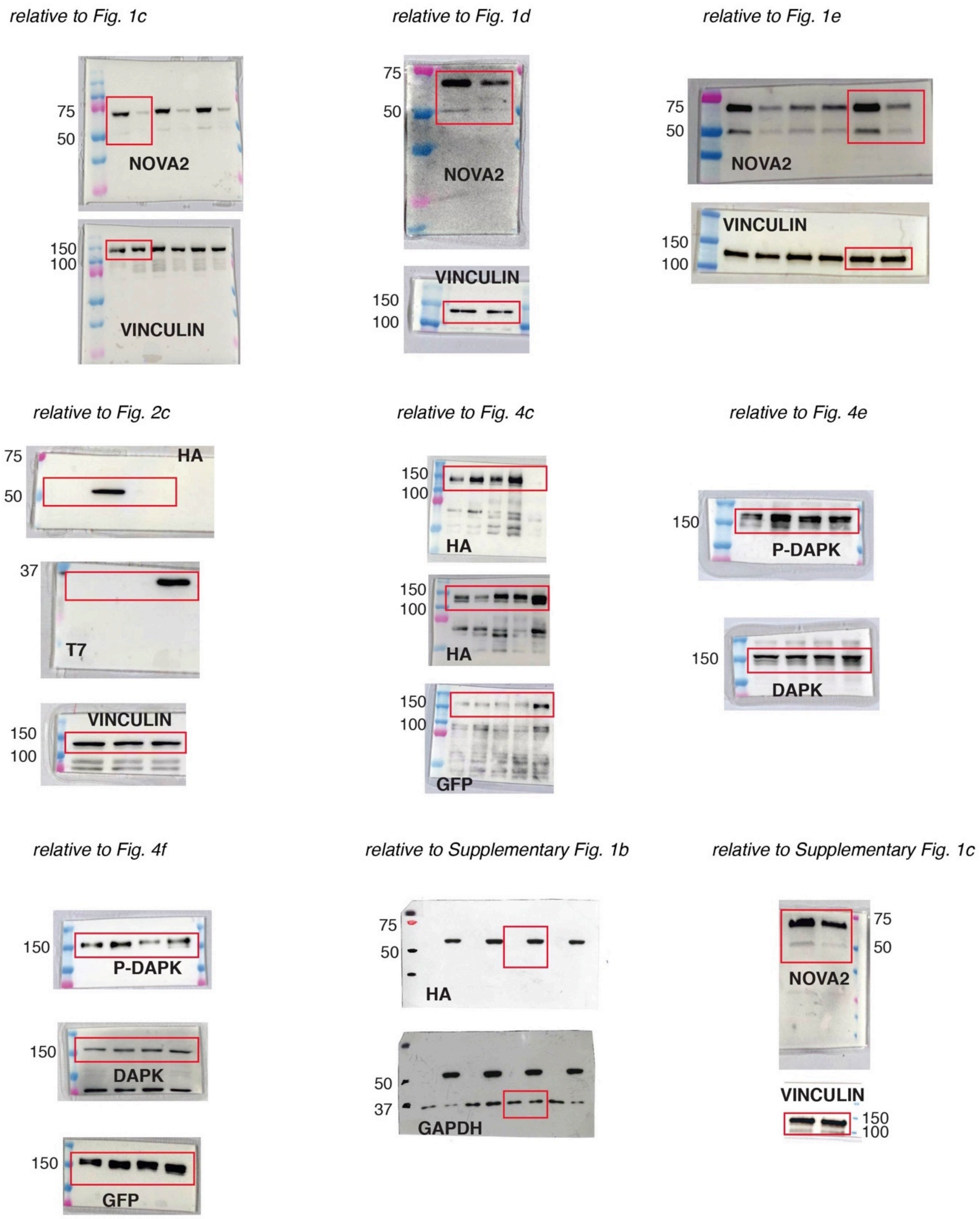

relative to Supplementary Fig. 2a

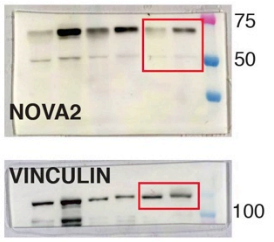

relative to Supplementary Fig. 2b

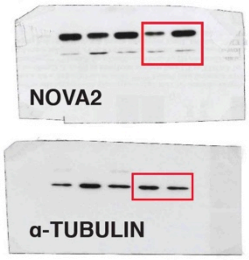

relative to Supplementary Fig. 2c

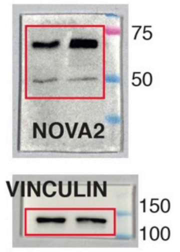

relative to Supplementary Fig. 4a

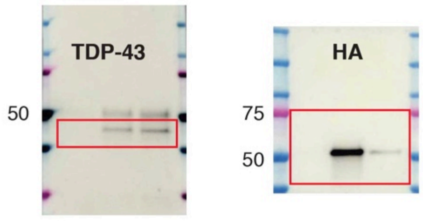

relative to Supplementary Fig. 4b

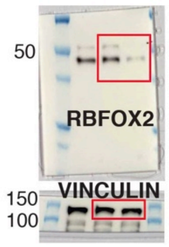

relative to Supplementary Figure 5c

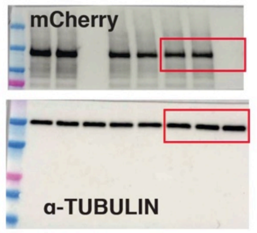

relative to Supplementary Figure 7b

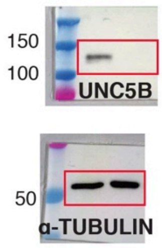

relative to Supplementary Figure 7d

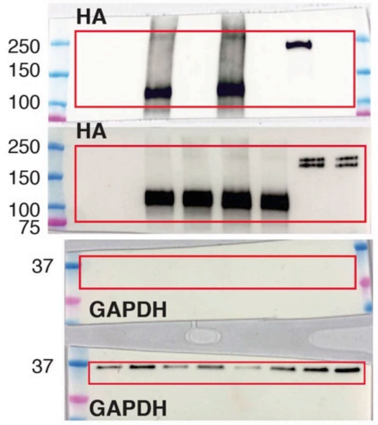

relative to Supplementary Figure 7f

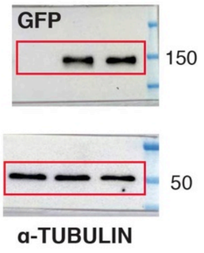

relative to Supplementary Fig. 8b

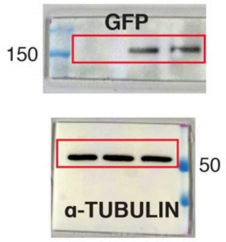

relative to Supplementary Fig. 8c

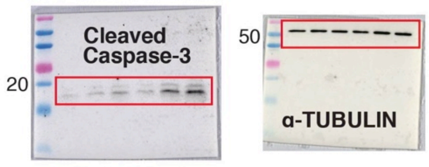

relative to Supplementary Fig. 8d

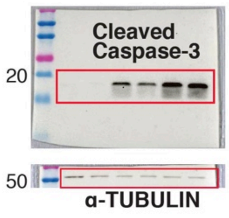

Supplementary Fig. 20. Uncropped original RT-PCR gels.

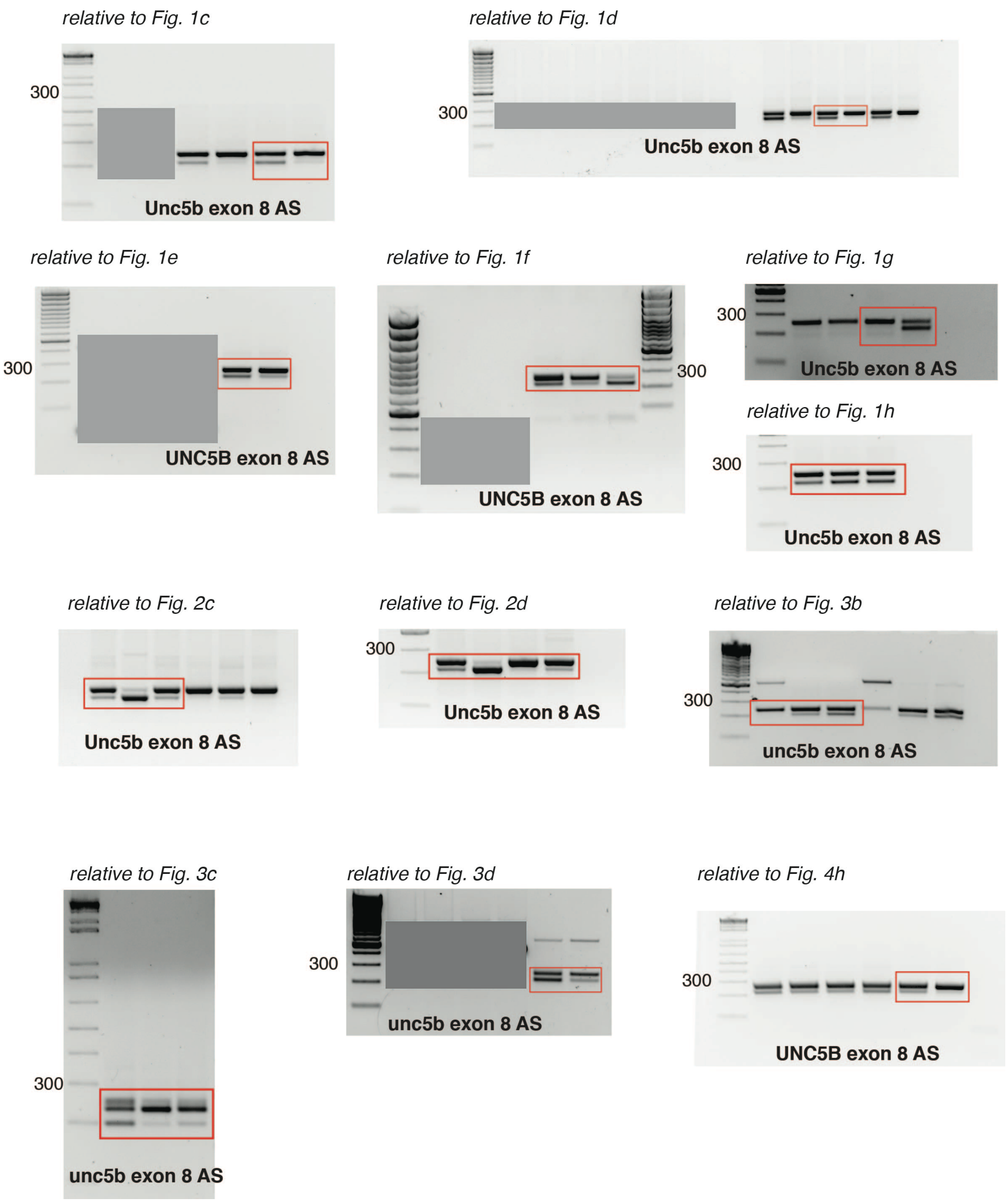

relative to Supplementary Fig. 1b

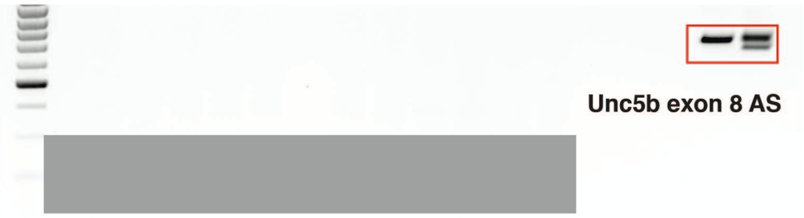

relative to Supplementary Fig. 1c

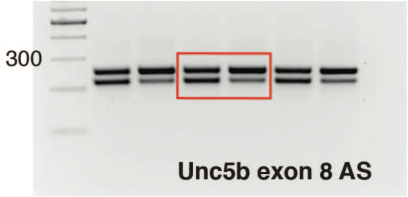

relative to Supplementary Fig. 4b, 1d

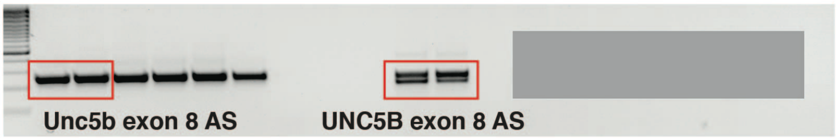

relative to Supplementary Fig. 2a

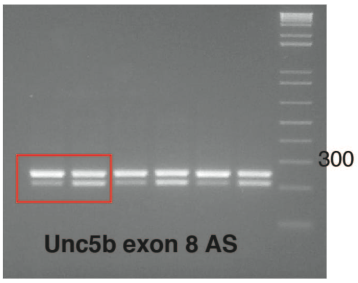

relative to Supplementary Fig. 5b

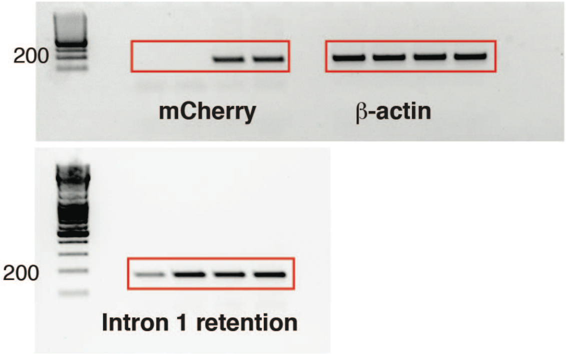

relative to Supplementary Fig. 2b

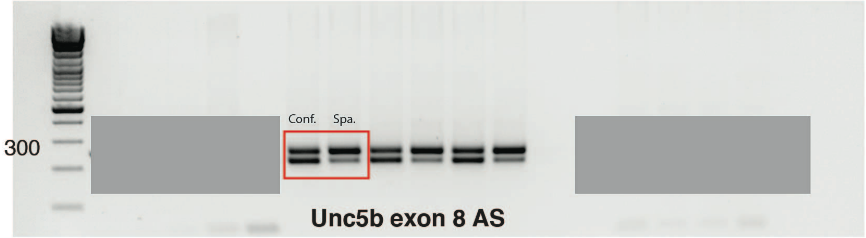

relative to Supplementary Fig. 2c

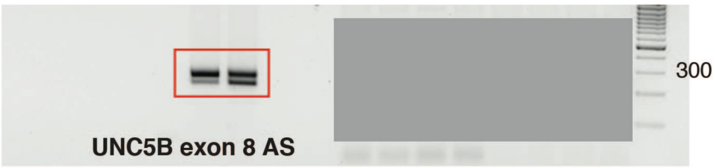

relative to Supplementary Fig. 10b

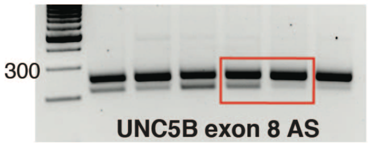

relative to Supplementary Fig. 13b

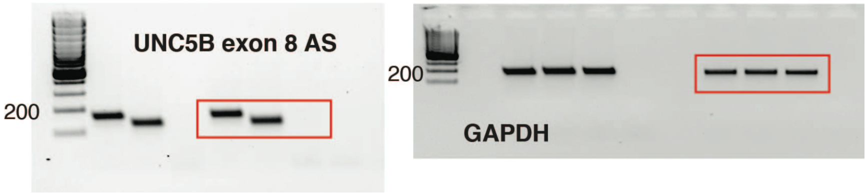

relative to Supplementary Fig. 14c

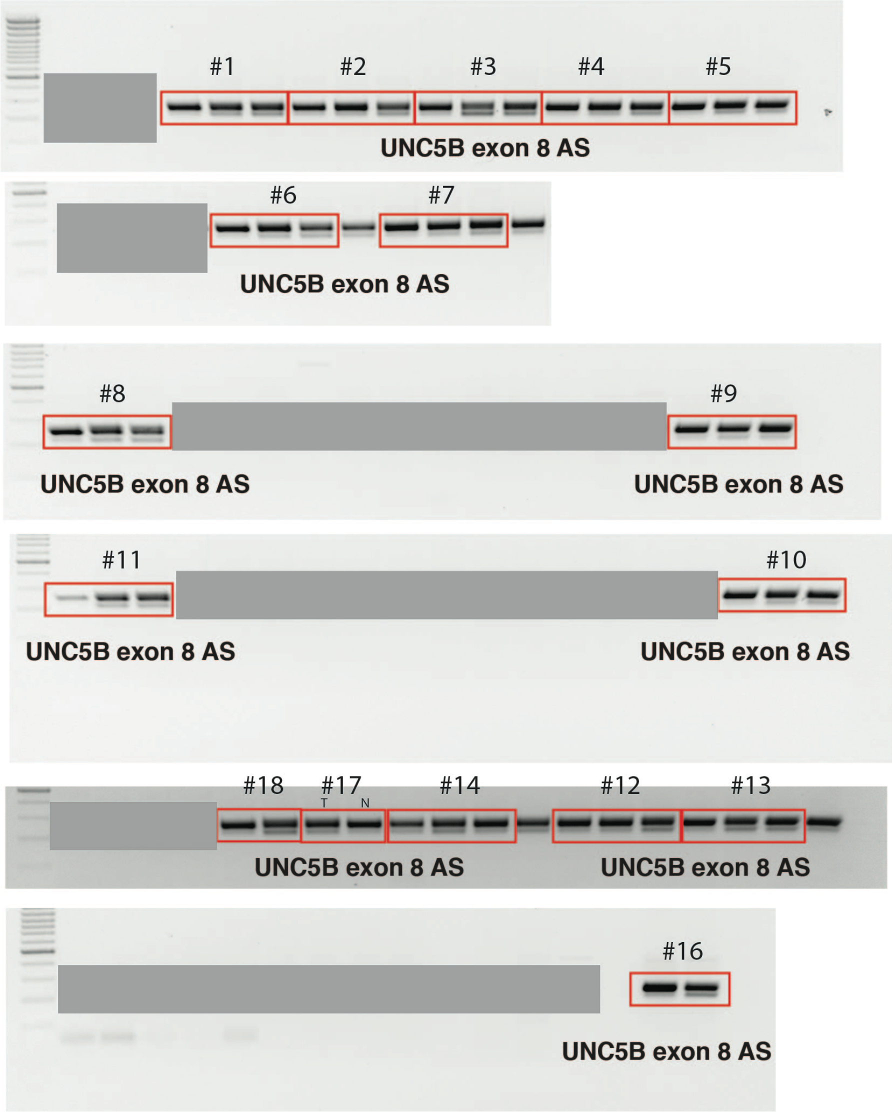

Supplementary Table 1. Primers used in RT-PCR, RT-qPCR, and cloning experiments.

Primer name	Sequence (5'→3')
**RT-PCR experiments:**	
mouse-Unc5b_E8-F	CCAAGAACTGCACTGATGGG
mouse-Unc5b_E8-R	GAAGTTGACAGGGTGGAAGC
human-UNC5B_E8-F	GACGGAGTGGAGCAAGTGGT
human-UNC5B_E8-R	ACGAAGATGGCCACCACGAG
human-UNC5B_E8-F_shift	GCGTAGCCGCGAGTGCAT
human-UNC5B_E8-R_shift	GCATCCCCTGAGGCCTC
zebrafish-unc5b_E8-F	TCGTGAATGTCAGGCTCCAC
zebrafish-unc5b_E8-R	CGGCGATAGACCAGAATCCC
zebrafish-unc5b_i1-F	GGAGTCGCACTGTCTGGATT
zebrafish-unc5b_i1-R	CCGCCCACTGTTCTATGAGC
mCherry-F	CAGCTCTGGAAGAGATGGGC
mCherry-R	CTCCATGTGCACCTTGAAGC
zebrafish-actb-F	ACATCAGGGTGTCATGGTTGGT
zebrafish-actb-R	CGCAGCTCGTTGTAGAAGGTGT
BGH-rev	TAGAAGGCACAGTCGAGG
**RT-qPCR experiments:**	
mouse-Nova2-F	AGGACTGATCATCGGTAAGG
mouse-Nova2-R	GGGTCTTCCTGTACCTTCTG
human-NOVA2-F	CAGCTTTATTGCCGAGAAGG
human-NOVA2-R	ACCCATGCTCCTGACTGTTC
zebrafish-nova2-F	CATTATTGGCAAGGGTGGTC
zebrafish-nova2-R	TGCAATGAAGTCGTGGACAT
human-COL4A1-F	CTACGTGCAAGGCAATGAACG
human-COL4A1-R	GCAGAACAGGAAGGGCATTGT
mouse/human-Gapdh-F	TCAAGAAGGTGGTGAAGCAGG
mouse/human-Gapdh-R	ACCAGGAAATGAGCTTGACAAA
mouse-Ubb-F	TCTTCGTGAAGACCCTGACC
mouse-Ubb-R	CAGGTGCAGGGTTGACTCTT
human-ACTB-F	AGAGCTACGAGCTGCCTGAC
human-ACTB-R	AGTTTCGTGGATGCCACAGG
human-B2M-F	AAGTGGAGCATTCAGACTTGT
human-B2M-R	CACGGCAGGCATACTCATCT
CLIP-Unc5b_A-F	AGCTGTAGGGCCTCTGTCTG
CLIP-Unc5b_A-R	GTGACAGAGAAAGGGTGGCC
CLIP-Unc5b_B-F	CTCCTTGGCTGGCTTTCCAT
CLIP-Unc5b_B-R	GCATGCATGAGGTACAGGGA
CLIP-Unc5b_C-F	GTGAGTGTGGAGCTGAGCTT
CLIP-Unc5b_C-R	AAGCCAGGAGGTGCTAGAGA
human-Unc5b-F	CTGGGACCTTATGCCTTCAA
human-Unc5b-R	CGCTTTGGTGGCAAAGTAAT
**Cloning experiments:**	
Unc5b-For-HindIII	CAAGCTTACCATGAGGGCCCGGAGCGGGGTGC
Unc5b-Rev-EcoRI-HA	CCGGAATTCTCAAGCGTAATCTGGAACATCGTATG GGTACATTCCGCAATCGCCATCTGTGGCCATG
Unc5b-Rev-EcoRI-GFP	CCGGAATTCGTCCGCAATCGCCATCTGTGGCCATG
Unc5b-deletion_E8-F	TGATGGGCTGTGCGTGCTGACCCTGGAGACATCGG GAGAT
Unc5b-deletion_E8-R	ATCTCCCGATGTCTCCAGGGTCAGCACGCACAGCCC ATCA
Unc5b_minigene_F	CCCGGATCCTGGATGGAGCGTGGACCGA
Unc5b_minigene_R	GGGGAATTCTGGGCCTTGCAGTCTTGAAG
Unc5b_minigene_mut_F	TGCAATGCCCCAATAACCTCCTCGGCAACCCTTTC TCTGTAACCTGAGTAATCATCTTTCCCTTTATTTC
Unc5b_minigene_mut_R	TGCCGAGGAGGTTATTGGGGCATTGCATTGATTAG ATTAAGGGCGAGCGTTGGGGCCAGACAGAGGCCC TA

Unc5b_zebrafish-F	GCCGGATCCGGTACCACGCGTGACCGGGTGAAGA TGCT
Unc5b_zebrafish-R	TTGGGATCCTCCAGTAAAACC
Unc5bEcto-deletion_E8-F	TGCTGGAGGCCTCAGGGGAT
Unc5bEcto-deletion_E8-R	TTTGCATGCACAGCCCATCT
***In vitro* RNA pull down:**	
T7_YCAYcluster_F	TAATACGACTCACTATAGGGCCTCTGTCTGGCCC
TDP43_YCAYcluster _R	CACACACACACACACACTAAAGGGAAAGATG

Supplementary Table 2. Morpholino antisense oligonucleotide.

Morpholino name	Sequence (5'→3')
zebrafish-MO-*unc5b*	CATTTAACCGGCTCGTACCTGCATG
human-MO-*i7_UNC5B*	AAGGATGAGATGAGATGATGGATGT
