# Supplementary material for: A ligand-insensitive UNC5B splicing isoform regulates angiogenesis by promoting apoptosis
Source: Nat Commun. 2021 Aug 11;12:4872. doi: 10.1038/s41467-021-24998-6 (PMC8358048; doi:10.1038/s41467-021-24998-6)
Supplement: Supplementary file 3 — Reporting Summary [file 41467_2021_24998_MOESM3_ESM.pdf]

nature research

Corresponding author(s): CLAUDIA GHIGNA

Last updated by author(s): Jul 5, 2021

Reporting Summary

Nature Research wishes to improve the reproducibility of the work that we publish. This form provides structure for consistency and transparency in reporting. For further information on Nature Research policies, see our Editorial Policies and the Editorial Policy Checklist.

Statistics

For all statistical analyses, confirm that the following items are present in the figure legend, table legend, main text, or Methods section.

n/a	Confirmed	
☐	☒	The exact sample size (*n*) for each experimental group/condition, given as a discrete number and unit of measurement
☐	☒	A statement on whether measurements were taken from distinct samples or whether the same sample was measured repeatedly
☐	☒	The statistical test(s) used AND whether they are one- or two-sided<br>*Only common tests should be described solely by name; describe more complex techniques in the Methods section.*
☒	☐	A description of all covariates tested
☐	☒	A description of any assumptions or corrections, such as tests of normality and adjustment for multiple comparisons
☐	☒	A full description of the statistical parameters including central tendency (e.g. means) or other basic estimates (e.g. regression coefficient) AND variation (e.g. standard deviation) or associated estimates of uncertainty (e.g. confidence intervals)
☐	☒	For null hypothesis testing, the test statistic (e.g. *F*, *t*, *r*) with confidence intervals, effect sizes, degrees of freedom and *P* value noted<br>*Give P values as exact values whenever suitable.*
☒	☐	For Bayesian analysis, information on the choice of priors and Markov chain Monte Carlo settings
☒	☐	For hierarchical and complex designs, identification of the appropriate level for tests and full reporting of outcomes
☐	☒	Estimates of effect sizes (e.g. Cohen's *d*, Pearson's *r*), indicating how they were calculated

Our web collection on statistics for biologists contains articles on many of the points above.

Software and code

Policy information about availability of computer code

Data collection

-Western blot: immunostained bands were detected under a ImageQuant LAS 500 (GE Healthcare);
-RT-qPCR: LightCycler 480 (Roche);
-RT-PCR: Gel Doc EZ Imaging System (Biorad), Image Lab version 5.2.1;
-Caspase-3 Activity assay: Synergytm HT Microplate reader (BioTeK Instruments);
-Zebrafish embryos images: Leica TCS SP2 confocal microscope;
-Surface Plasmon Resonance: BIAcore T200 (GE Healthcare);
-Cell imaging:Zeiss LSM800, Leica SP5 confocal microscope with a Leica spectral detection system (Leica 15 SP detector), or Optical Microscope Olympus IX71
-Digital microscopy: Aperio CS2 digital scanner and ScanScope software (Leica Biosystems; #12.03.5048)

Data analysis

-RT-qPCR: LightCycler 480 Software (Roche);
-RT-PCR AS quantification: ImageJ 1.48v;
-Surface Plasmon Resonance: BIAcore BIAevaluation software v. 3.0;
-Cell imaging: Zeiss Zen 2.3 Software, Leica application suite advanced fluorescence software , or MetaMorph 7.7.5 software (Universal Imaging Corporation);
-RBP motifs prediction was performed by using RBPmap web server (http://rbpmap.technion.ac.il; Paz et al., 2014) and SpliceAid 2 (http://www.introni.it/spliceaid.html; Piva et al., 2011).
-Gene expression and splicing analyses of transcriptomic data: UALCAN (http://ualcan.path.uab.edu; Chandrashekar et al., 2017); Oncomine (http://oncomine.org/resource/; Rhodes et al., 2004); TCGA SpliceSeq (http://projects.insilico.us.com/TCGASpliceSeq; Ryan et al., 2016); Human Cancer Metastasis Database (HCMDB) (https://hcmdb.i-sanger.com; Zheng et al., 2018); TSVdb web-tool (http://www.tsvdb.com; Sun et al., 2018); PROGgeneV2 tool (http://genomics.jefferson.edu/proggene; Goswami and Nakshatri, 2014); VastDB (https://vastdb.crg.eu/wiki/Main_Page; Tapial et al., 2017);
-Identification of intrinsically disordered regions (IDR): GlobPlot prediction analysis (http://globplot.embl.de; Linding et al., 2003) and PONDR prediction (http://www.pondr.com);

-Imaging analysis: Adobe Photoshop Version 14.1.2.x64;
-For statistical analysis and graphic representation: GraphPad Prism v6;

For manuscripts utilizing custom algorithms or software that are central to the research but not yet described in published literature, software must be made available to editors and reviewers. We strongly encourage code deposition in a community repository (e.g. GitHub). See the Nature Research guidelines for submitting code & software for further information.

Data

Policy information about availability of data

All manuscripts must include a data availability statement. This statement should provide the following information, where applicable:

- Accession codes, unique identifiers, or web links for publicly available datasets
- A list of figures that have associated raw data
- A description of any restrictions on data availability

Previously published RNA-sequencing data that were re-analysed here are available under accession code BioProject PRJNA293346 (Giampietro et al., 2015). All gene and transcripts data, patient's survival, and clinical information that support the findings of this study are available in GDC Data Portal (https://portal.gdc.cancer.gov/projects/TCGA-COAD), ONCOMINE (http://www.oncomine.org), HCMDB databases (https://hcmdb.i-sanger.com), PROGgeneV2 tool (http://genomics.jefferson.edu/proggene) and TSVdb web-tool (http://www.tsvdb.com). The authors declare that all other data supporting the findings of this study are available within the paper (and its supplementary information files).

Field-specific reporting

Please select the one below that is the best fit for your research. If you are not sure, read the appropriate sections before making your selection.

☒ Life sciences ☐ Behavioural & social sciences ☐ Ecological, evolutionary & environmental sciences

For a reference copy of the document with all sections, see nature.com/documents/nr-reporting-summary-flat.pdf

Life sciences study design

All studies must disclose on these points even when the disclosure is negative.

Sample size	Sample sizes were estimated on the basis of previous experiments conducted in our laboratory providing a sufficient numerosity for each group to yield a two-sided statistical test that can reach a power of 0.9 (subject to alpha= 0.05).
Data exclusions	No data were excluded for pre-established criteria.
Replication	All experiments were reproduced at least three independent times. To verify their reproducibility the experiments were successfully repeated using different lots of each reagents, different stocks of cell lines and, for some experiments, were repeated and confirmed by different operators.
Randomization	Zebrafish embryos were randomly assigned to the different experimental groups. For in vitro experiments, samples were randomly treated according to the experimental design.
Blinding	For in vivo and in vitro experiments during the different procedures blinding to group allocation and analysis was not possible since embryos or cells need to be treated with different reagents.

Reporting for specific materials, systems and methods

We require information from authors about some types of materials, experimental systems and methods used in many studies. Here, indicate whether each material, system or method listed is relevant to your study. If you are not sure if a list item applies to your research, read the appropriate section before selecting a response.

Materials & experimental systems

n/a	Involved in the study	
☐	☒	Antibodies
☐	☒	Eukaryotic cell lines
☒	☐	Palaeontology and archaeology
☐	☒	Animals and other organisms
☐	☒	Human research participants
☒	☐	Clinical data
☒	☐	Dual use research of concern

Methods

n/a	Involved in the study	
☒	☐	ChIP-seq
☒	☐	Flow cytometry
☒	☐	MRI-based neuroimaging

Antibodies

Antibodies used

The following primary antibodies were used for immunoblot analyses: anti-NOVA2 (C-16) (1:200; Santa Cruz Biotechnology, #sc-10546), anti-a-TUBULIN (1:100,000 Sigma-Aldrich; #T9026), anti-haemagglutinin (HA; 1:1,000 Roche; #11867423001), anti-GAPDH (1:5,000 Abcam; #ab75834), anti-GFP (1:3,000 Millipore; #MAB3580); anti-Cleaved Caspase-3 (1:1,000 Cell Signaling; #9661); anti-VINCULIN (1:5,000 Millipore; #MAB3574); anti-UNC5B (1:1,000 Cell Signaling: #13851); anti-DAPK (pSer308) (1:1,000 Sigma-Aldrich; #D4941); Anti-DAPK (1:1000 Sigma-Aldrich; #D1319); anti-T7 (1:1000 Novagen; #69522-3); anti-RBFOX2 (1:2,000 Bethyl Laboratories; #A300-864A); anti-mCherry (1:1,000 Chromotek; #5f8); TDP-43 (1:1000 Proteintech; #10782-2-AP).
The following primary antibodies were used for immunohistochemistry analyses: anti-NOVA2 (1:100, Sigma-Aldrich; HPA045607); anti-CD31 (clone PECAM-1, 1:100, Leica); anti-Cleaved Caspase-3 (polyclonal rabbit, 1:600, R&D Systems).
The following secondary antibodies were used for immunohistochemistry analyses: Peroxidase AffiniPure Goat Anti-Mouse IgG (H+L) (1:5000, Jakson Immuno Research, 115-035-146); Peroxidase AffiniPure Donkey Anti-Rabbit IgG (H+L) (1:10000, Jakson Immuno Research, 711-035-152); Peroxidase AffiniPure Donkey Anti-Goat IgG (H+L) (1:5000, Jakson Immuno Research, 705-035-147); Peroxidase AffiniPure Goat Anti-Rat IgG, light chain specific (1:5000, Jakson Immuno Research, 112-035-175); Goat anti-Rat IgG (H+L) Cross-Adsorbed Secondary Antibody, Alexa Fluor 633 (1:400, ThermoFisher Scientific, A-21094); Donkey anti-Mouse IgG (H+L) Highly Cross-Adsorbed Secondary Antibody, Alexa Fluor Plus 488 (1:400, ThermoFisher Scientific, A-32766).

Validation

All antibodies are commercially available. Validation for species and application was provided by the manufactures or by others as reported below:
anti-NOVA2 (C-16): Saito et al., 2016;
anti-a-TUBULIN: Independent Antibody Verification;
anti-haemagglutinin: Function tested in western blot;
anti-GAPDH: Abpromise guarantee
anti-GFP: 100% Performance Guaranteed validated antibodies;
anti-Cleaved Caspase-3 (Cell Signaling): WB validation provided by Cell Signaling;
anti-VINCULIN: 100% Performance Guaranteed validated antibodies;
anti-UNC5B: WB validation provided by Cell Signaling;
anti-DAPK (pSer308): validation provided by Sigma-Aldrich;
anti-DAPK: validation provided by Sigma-Aldrich;
anti-T7: The antibody was tested for specific reactivity with a 31,102 dalton target protein in the T7•Tag positive control extract on dot blots and Western blots;
anti-RBFOX2: validation provided by Bethyl Laboratories;
anti-mCherry: Tested on dsRed, mRFP, mCherry , mPlum, mRFPruby, mScarlet, tdTomato;
TDP-43: validation provided by Proteintech;
anti-NOVA2 (HPA045607): orthogonal RNAseq Antibody Verification;
anti-CD31: In Vitro Diagnostic Use;
anti-Cleaved Caspase-3 (R&D Systems): validation provided by R&D Systems.
All antibodies are commercially available. Validation for species and application was provided by the manufactures or by others as reported below:
anti-NOVA2 (C-16): Saito et al., 2016;
anti-a-TUBULIN: Independent Antibody Verification;
anti-haemagglutinin: Function tested in western blot;
anti-GAPDH: Abpromise guarantee
anti-GFP: 100% Performance Guaranteed validated antibodies;
anti-Cleaved Caspase-3 (Cell Signaling): WB validation provided by Cell Signaling;
anti-VINCULIN: 100% Performance Guaranteed validated antibodies;
anti-UNC5B: WB validation provided by Cell Signaling;
anti-DAPK (pSer308): validation provided by Sigma-Aldrich;
anti-DAPK: validation provided by Sigma-Aldrich;
anti-T7: The antibody was tested for specific reactivity with a 31,102 dalton target protein in the T7•Tag positive control extract on dot blots and Western blots;
anti-RBFOX2: validation provided by Bethyl Laboratories;
anti-mCherry: Tested on dsRed, mRFP, mCherry , mPlum, mRFPruby, mScarlet, tdTomato;
TDP-43: validation provided by Proteintech;
anti-NOVA2 (HPA045607): orthogonal RNAseq Antibody Verification;
anti-CD31: In Vitro Diagnostic Use;
anti-Cleaved Caspase-3 (R&D Systems): validation provided by R&D Systems.
anti-RBFOX. Bethyl Lab RBFOX oratories, #A300-864A

Eukaryotic cell lines

Policy information about cell lines

Cell line source(s)

Mouse embryonic ECs (moEC), previously referred as vascular endothelial (VE) cadherin-positive ECs, were described in Taddei et al., 2008 and Lampugnani et al., 2002. Mouse lung-derived luEC and lu2EC were described in Magrini et al., 2014 and Bazzoni et al., 2015, respectively. Primary human umbilical vein endothelial cells (HUVECs) were obtained from either Yale University Vascular Biology and Therapeutics Core Facility or purchased (Lonza, #CC-2519). Immortalized HUVEC (HUVEC/TERT2) were purchased from Evericyte. HeLa and HEK-293A cells were purchased from ATCC. FreeStyle™ HEK293-F cells were purchased from Life Technologies.

Authentication

moEC, luEC, and lu2EC characterization was described in Lampugnani et al., 2002, Magrini et al., 2014, and Bazzoni et al.,

Authentication	2015. HUVEC and HUVEC/TERT2 were verified by CD31 and VE-cadherin staining for endothelial cell identify. HeLa (ATCC), HEK-293A (ATCC), and Freestyle''' HEK293-F were authenticated by the respectively vendors (ATCC: STR profiling).
Mycoplasma contamination	All cells were free of mycoplasma contamination.
Commonly misidentified lines (See ICLAC register)	None

Animals and other organisms

Policy information about studies involving animals; ARRIVE guidelines recommended for reporting animal research

Laboratory animals	Zebrafish from wild-type AB, transgenic Tg(Kdrl:GFP)la116 (Cross et al., 2003), Tg(flila:GFP)y1 (Lawson and Weinstein, 2002), and Tg(kdrl:GFP)la116;nova2io011 (Giampietro et al., 2015). Zebrafish male and female embryos were analyzed from 24 hours to 6 day post-fertilization.
Wild animals	The study did not involve wild animals
Field-collected samples	The study did not involve field-collected sample
Ethics oversight	All animal strains were maintained and bred according with standard procedures and national guidelines (Italian decree “4th March 2014, n.26”). All experiments were approved by the Italian Ministry of Health, and were performed under the supervision of the institutional organism for animal welfare (Cogentech OPBA).

Note that full information on the approval of the study protocol must also be provided in the manuscript.

Human research participants

Policy information about studies involving human research participants

Population characteristics	TCGA-COAD patient information, including gender, mutational status and clinical data, could be retrieved through GDC Data Portal (https://portal.gdc.cancer.gov/projects/TCGA-COAD).<br>Protocol using human material were approved by the respective medical and scientific committee. No covariate-relevant population characteristic were present for the analysis of UNC5B and NOVA2 expression in tumors versus normal tissues.<br>Human primary colorectal cancer, adjacent normal colorectal and liver metastasis snap-frozen tissue samples were obtained from Biological Resource Centre (CRB, Centre de Resource Biologique) of Léon Bérard cancer Centre (Lyon, France) (protocol number: BB-0033-00050), as previously described (Di Ruocco, et al., 2018).<br>TMA comprised normal and neoplastic areas for each case and were generated from archival tissue blocks retrieved from the Department of Pathology (ASST-Spedali Civili di Brescia, Brescia, Italy).
Recruitment	TCGA-COAD patient recruitment information could be retrieved through GDC Data Portal (https://portal.gdc.cancer.gov/projects/TCGA-COAD). Recruitment information of the different datasets used in this study are available through access to the original studies.
Ethics oversight	Sequencing of human subjects' tissue was performed by TCGA consortium members under a series of locally approved Institutional Review Board (IRB) protocols as described in (Cancer Genome Atlas Network (2012)). Informed consent was obtained from all human participants.<br>For RNA extracted from Human primary colorectal cancer, adjacent normal colorectal and liver metastasis snap-frozen tissue CRB medical and scientific committee approved the protocol (Biological Resource Centre of Leon Berard cancer Centre, Lyon, France). All patients signed informed consent to participate to research according to the French laws.<br>Clinical samples were represented by a multitumor TMA (including cores from 126 primary carcinomas) and paraffin tissue blocks from archival colorectal carcinomas were retrieved from the archive of Pathology (U.O. Anatomia Patologica, Spedali Civili di Brescia). The study supervised by WV, was approved by the local IRB (code: WW-IMMUNOCANCERhum, NP-906).

Note that full information on the approval of the study protocol must also be provided in the manuscript.
